# Endothelial dysfunction and immunothrombosis in sepsis

**DOI:** 10.3389/fimmu.2023.1144229

**Published:** 2023-04-04

**Authors:** Eleni Maneta, Evmorfia Aivalioti, Simon Tual-Chalot, Besa Emini Veseli, Aikaterini Gatsiou, Kimon Stamatelopoulos, Konstantinos Stellos

**Affiliations:** ^1^ Department of Clinical Therapeutics, National and Kapodistrian University of Athens Medical School, Athens, Greece; ^2^ Biosciences Institute, Vascular Biology and Medicine Theme, Faculty of Medical Sciences, Newcastle University, Newcastle Upon Tyne, United Kingdom; ^3^ Department of Cardiovascular Research, European Center for Angioscience (ECAS), Heidelberg University, Mannheim, Germany; ^4^ Translational and Clinical Research Institute, Vascular Biology and Medicine Theme, Faculty of Medical Sciences, Newcastle University, Newcastle upon Tyne, United Kingdom; ^5^ German Centre for Cardiovascular Research (DZHK), Partner Site Heidelberg/Mannheim, Mannheim, Germany; ^6^ Department of Cardiology, University Hospital Mannheim, University of Heidelberg, Mannheim, Germany

**Keywords:** immunothrombosis, endothelial dysfunction, neutrophil extracellular traps, tissue factor, sepsis

## Abstract

Sepsis is a life-threatening clinical syndrome characterized by multiorgan dysfunction caused by a dysregulated or over-reactive host response to infection. During sepsis, the coagulation cascade is triggered by activated cells of the innate immune system, such as neutrophils and monocytes, resulting in clot formation mainly in the microcirculation, a process known as immunothrombosis. Although this process aims to protect the host through inhibition of the pathogen’s dissemination and survival, endothelial dysfunction and microthrombotic complications can rapidly lead to multiple organ dysfunction. The development of treatments targeting endothelial innate immune responses and immunothrombosis could be of great significance for reducing morbidity and mortality in patients with sepsis. Medications modifying cell-specific immune responses or inhibiting platelet–endothelial interaction or platelet activation have been proposed. Herein, we discuss the underlying mechanisms of organ-specific endothelial dysfunction and immunothrombosis in sepsis and its complications, while highlighting the recent advances in the development of new therapeutic approaches aiming at improving the short- or long-term prognosis in sepsis.

## Introduction

The latest definition by the Third International Consensus Definition Task Force defines sepsis as a life-threatening organ dysfunction resulting from a dysregulated host response to infection (1). Despite a better understanding of the underlying pathophysiological mechanisms and improvements to existing therapeutic algorithms, morbidity and mortality in patients with sepsis still remain high. It is estimated that 2.8 million deaths per year are attributed to sepsis, with one in three hospital deaths related to it (2, 3). Sepsis can originate from any infecting organism, but the most frequently identified ones are *Staphylococcus aureus* (*S. aureus*), *Pseudomonas* species, and *Escherichia coli* (*E. coli*). Among different sites susceptible to infection, the main ones leading to sepsis are the lungs (64%), the abdomen (20%), the bloodstream (15%), and the renal and genitourinary tract (14%) (2).

Activated components of innate immunity act as the first line of host immune defense against invading pathogens (4). Apart from their direct role in clearing the microorganism, neutrophils, monocytes, and the complement can interact with platelets, and the coagulation cascade leading to the local formation of thrombi in microvessels. This process, known as “immunothrombosis,” contributes to the recognition and restriction of a pathogen’s spread, tissue invasion, and survival, thus consisting an essential component of intravascular immunity (5). Although this distinct innate immune mechanism seems initially beneficial for the host, uncontrolled activation of this process in sepsis can trigger aberrant thrombosis in the microcirculation. During immunothrombosis, immune cells interact also with endothelial cells, which acquire a proadhesive and prothrombotic phenotype (5). Indeed, coagulation abnormalities are common in patients with sepsis, with severity ranging from subclinical coagulation activation to devastating thrombotic and hemorrhagic complications (6). In sepsis, both the activation of the coagulation cascade and the dysregulation of the endogenous anticoagulant and fibrinolytic mechanisms lead to microvascular thrombosis and tissue ischemia, which in turn contribute to tissue injury and multiorgan failure (7). Specifically, high thrombin and d-dimer plasma levels, both markers of coagulation activation, are considered indicative of severe sepsis (8, 9). Accordingly, decreased plasma levels of natural anticoagulants, such as protein C, protein S, and antithrombin (AT), have been found in sepsis and are associated with worse disease prognosis (9–11). Disseminated intravascular coagulation (DIC) is estimated to occur in up to 30%–50% of patients with severe sepsis, and it is associated with a twofold higher mortality risk (12). DIC is characterized by endothelial dysfunction and dysregulation of procoagulant, anticoagulant, and fibrinolytic factors (8). In sepsis-associated DIC, immunothrombosis is the underlying pathophysiological mechanism that triggers the initiation of DIC (5). Most of our knowledge regarding immunothrombosis suggests that it is a process mainly taking place in the microvasculature, affecting both arterioles and venules.

Despite the high prevalence of coagulation abnormalities and their potential deleterious effect on the outcome of sepsis patients, the current treatment approach is generally focused on antibiotics, fluid resuscitation, vasopressors, as well as oxygen and ventilatory support (13). Apart from heparin’s use for venous thromboembolism (VTE) prophylaxis in patients with sepsis, effective treatments specifically targeting the process of immunothrombosis, aiming to abrogate the devastating complications while maintaining its beneficial effects for the host, do not yet exist (13). A better understanding of the underlying mechanisms and cellular interactions in sepsis-related immunothrombosis is crucial to identifying new therapeutic targets that would open the field for the development of new treatments in sepsis. Our study summarizes the current literature regarding the role of endothelial cells and innate immune cells in immunothrombosis in sepsis and discusses clinical and preclinical evidence on drugs targeting immunothrombosis in sepsis.

## Overview of hemostasis and thrombosis

The process of thrombus formation inside a blood vessel, which can lead to partial or total vessel occlusion, requires platelets’ accumulation and activation as well as activation of the coagulation cascade (14). The binding of platelet glycoprotein Ib (GP Ib) receptor to von Willebrand factor (vWF) and the interaction of platelet glycoprotein IIb/IIIa (GP IIb/IIIa) receptor with fibrinogen are essential steps for platelet adhesion and aggregation, respectively (14). The coagulation cascade involves the activation of a series of clotting factors and is classically divided into three pathways: the extrinsic, the intrinsic, and the common pathway. The extrinsic pathway is initiated when tissue factor (TF) from the damaged vessel wall interacts with activated clotting factor VII (aFVII), while the intrinsic pathway begins when factor XII (FXII) interacts with the exposed collagen and becomes activated (aFXII). Both pathways end up in factor X (FX) activation, which in turn, along with activated factor V (aFV), cleaves prothrombin into thrombin. Afterward, thrombin activates fibrinogen into fibrin and also cleaves factor XIII (FXIII) into activated factor XIII (aFXIII), resulting in clot stabilization (common pathway) (15, 16). In normal hemostasis, along with the coagulation cascade activation, endogenous anticoagulant mechanisms are being recruited in order to prevent uncontrolled clot expansion and thrombosis. Tissue factor pathway inhibitor (TFPI), AT, activated protein C (APC), and protein S, as well as thrombomodulin (TM), which along with endothelial protein C receptor (EPCR) catalyzes the thrombin-mediated protein C activation, are some of the main physiologic anticoagulant mechanisms (17–20). Finally, under normal conditions, plasminogen is activated by tissue plasminogen activator (tPA) into plasmin, which catalyzes clot dissolution and fibrinolysis, while plasminogen activator inhibitor (PAI) serves as a tPA inhibitor (21, 22).

## Endothelial dysfunction in sepsis

### Signals leading to endothelial activation

**Figure 1:**
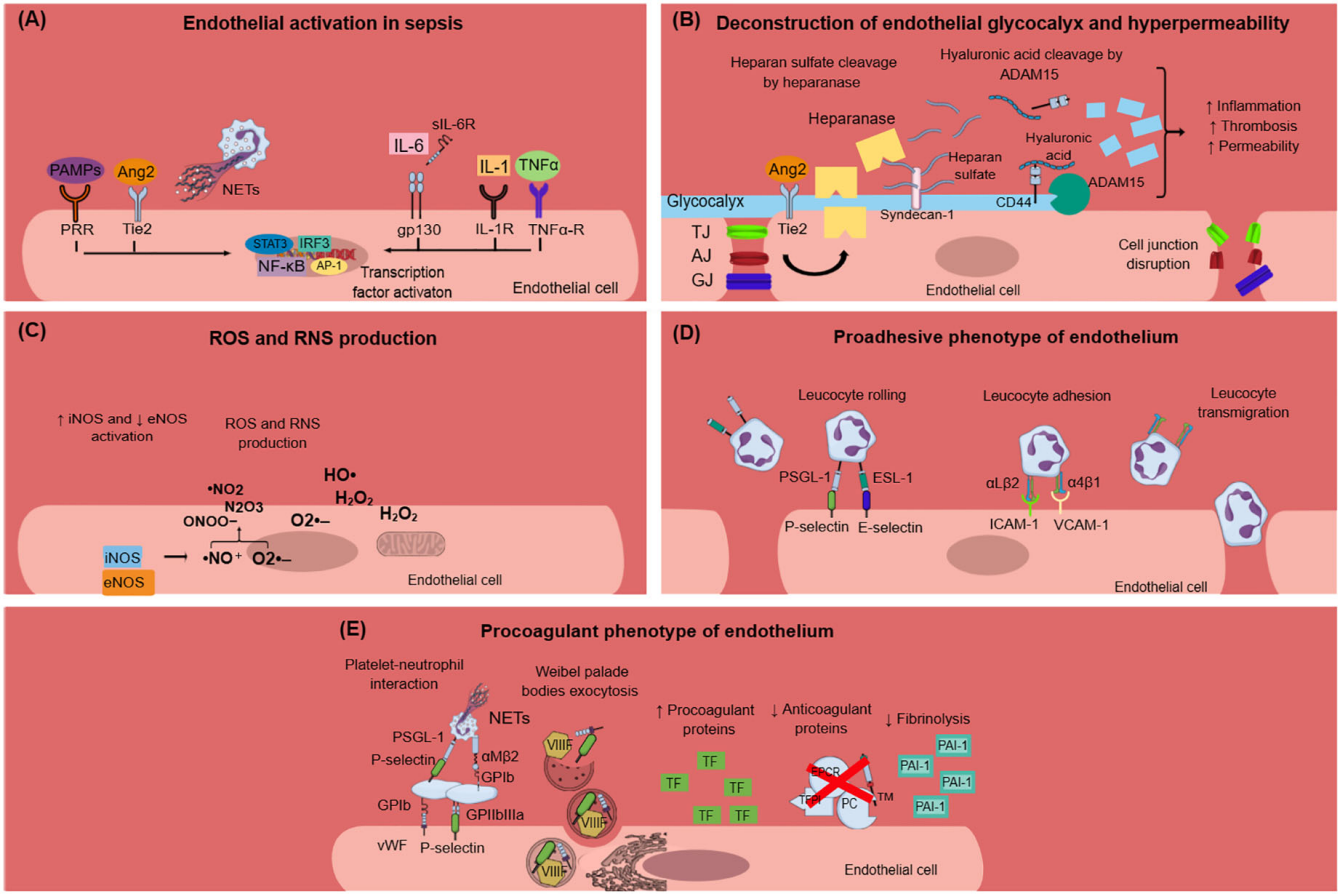
Endothelial dysfunction in sepsis. **(A)** PAMPs, TNF-α, IL-1, IL-6, Ang2, and NETs activate endothelial cells during sepsis. This results in intracellular signaling activation, leading to transcription factors activation, and mainly NF-κB. **(B)** Endothelial glycocalyx damage during sepsis is mediated through heparanase and ADAM15, which cleave heparan sulfate and CD44, respectively. Glycocalyx deconstruction results in increased inflammation, thrombosis, and hyperpermeability in sepsis. Furthermore, sepsis leads to endothelial cell junctions’ disruption, which subsequently leads to increased endothelial permeability. **(C)** Sepsis is characterized by dysregulated nitric oxide synthase activation, leading to the production of reactive nitrogen species. Moreover, infection triggers endothelial cells to produce ROS, which in turn destroy the endothelium. **(D)** Endothelium activation during sepsis results in the expression of E-selectin, P-selectin, VCAM-1, and ICAM-1 on the endothelial cell surface and subsequent leukocyte rolling, adhesion, and transmigration through endothelial cells. Leukocytes express specific ligands for binding with endothelial receptors such as PSGL-1 and ESL-1 for leukocyte rolling and αLβ2 and α4β1 integrins for leucocyte adhesion. **(E)** Activated endothelial cells during sepsis express molecules such as vWF and P-selectin on their surface, which mediate platelet aggregation. This leads to the formation of platelet-neutrophil aggregates and subsequent neutrophil activation. Weibel–Palade bodies contain procoagulant factors such as VIIIF, vWF, and P-selectin that can undergo exocytosis under sepsis conditions. The procoagulant phenotype of the endothelium is further characterized by increased production of TF and subsequent extrinsic coagulation pathway activation, decreased production of anticoagulant proteins (TFPI, EPCR, TM, PC), and increased production of PAI-1, which inhibits fibrinolysis. Ang2, angiopoietin 2; ADAM-15; disintegrin and metalloproteinase 15; AJ, adherens junctions; AP-1, activation of activator protein-1; eNOS, endothelial nitric oxide synthase; ESL-1, E-selectin ligand; GJ, gap junctions; gp130, glycoprotein 130; H_2_O_2_, hydrogen peroxide; HO·, hydroxyl radical; ICAM-1, intercellular adhesion molecule 1; IL, interleukin; IL-1R, interleukin-1 receptor; iNOS, inducible nitric oxide synthase; IRF3, interferon regulatory transcription factor-3; NETs, neutrophil extracellular traps; NF-κB, nuclear factor kappa-light-chain-enhancer of activated B cells; ·NO, nitric oxide; ·NO_2_, nitrogen dioxide; N_2_O_3_, dinitrogen trioxide; O_2_·–, superoxide anion; ONOO−, peroxynitrite; PAI-1, plasminogen activator inhibitor-1; PAMPs, pathogen associated molecular patterns; PC, protein C; PRR, pattern recognition receptor; PSGL-1, P-selectin glycoprotein ligand-1; RNS, reactive nitrogen species; ROS, reactive oxygen species; sIL-6R: soluble interleukin-6 receptor; STAT3, signal transducer and activator of transcription 3; TF, tissue factor; TFPI, tissue factor pathway inhibitor; TJ, tight junctions; TNF-α, tumor necrosis factor alpha; TNF-α-R, TNF-α receptor; VIIIF, VIII factor; VCAM-1, vascular cell adhesion molecule 1; vWF, von Willebrand factor; WPBs, Weibel–Palade bodies.

Many of the clinical manifestations related to sepsis can be attributed to endothelial dysfunction which constitutes one of the most prominent underlying pathophysiological mechanisms of sepsis and sepsis-related complications. Although a variety of inflammatory diseases are characterized by endothelial dysfunction, sepsis is linked to a distinctive procoagulant, proadhesive and apoptotic profile of endothelial cells (23). Normal endothelium contributes to vascular health through the expression of antithrombotic and anti-inflammatory molecules (23, 24). In sepsis, the endothelium is activated directly by pathogen-associated molecular patterns (PAMPs) of bacteria, viruses, and fungi or indirectly *via* neutrophil extracellular traps (NETs) and proinflammatory cytokines such as tumor necrosis factor-alpha (TNF-α), interleukin-6 (IL-6), and interleukin-1 (IL-1) (**Figure 1A**) (24–27). NETs are extracellular structures that are composed of cell-free DNA (cfDNA), histones, and antimicrobial proteins and are primarily released through a cell death process called NETosis or alternatively, through a process that does not require cell death, named non-lytic NETosis (28). PAMPs activate endothelial cells *via* pattern recognition receptors (PRRs), among which toll-like receptors (TLRs) exert the most profound inflammatory response in sepsis (29). Interestingly, endothelial activation during sepsis, as indicated by high circulating levels of intercellular adhesion molecule-1 (ICAM-1), vascular cell adhesion molecule-1 (VCAM-1), E-selectin, P-selectin, and vWF, is correlated with disease severity and mortality (30–32).

### Endothelial barrier dysfunction

One of the first signs of endothelial dysfunction in sepsis is endothelial glycocalyx (eGC) deconstruction (33) (**Figure 1B**). The endothelial glycocalyx is a carbohydrate-rich layer that covers the luminal surface of endothelial cells and serves numerous protective roles for the vascular system (34, 35). Specifically, eGC regulates vascular permeability and endothelial response to shear stress and inhibits leucocyte and platelet adhesion as well as coagulation cascade activation and hence microthrombus formation (36). During sepsis, distinctive mechanisms have been identified that promote eGC damage, which subsequently enhances endothelial dysfunction and sepsis complications. *In vivo* sepsis models suggest that sepsis-induced reduction in eGC thickness leads to increased albumin extravasation and circulating glycocalyx products such as hyaluronic acid and CD44 (37). The underlying mechanism can be explained by a cascade of reactions where disintegrin and metalloproteinase 15 (ADAM15) cleave CD44 of eGC, leading to an increase in plasma levels of soluble CD44 and hyaluronic acid. This is followed by a decrease in endothelial barrier integrity and a disruption of vascular endothelial (VE)-cadherin and β-catenin, which are essential components of adherens cell junctions (37). Similarly, endothelial tight and gap junctions were found to be disrupted in sepsis (38). A variety of eGC products are emerging as biomarkers in sepsis as they add important prognostic value. For example, increased syndecan-1 (SYN-1) plasma levels during sepsis are correlated with the requirement for renal replacement therapy, respiratory failure, multiple organ dysfunction syndrome (MODS), and predict coagulation failure and mortality (39–41). Sphingosine-1-phosphate (S1P) is another eGC component that predicts sepsis coagulation failure and mortality (40), while increased plasma endocan levels, another marker of eGC destruction, are also associated with the need for mechanical ventilation and with higher mortality in sepsis patients (42, 43). Angiopoietin-2 (Ang-2) is a growth factor produced by the endothelium under inflammatory conditions. It has been linked to increased vascular permeability in sepsis through enhancing endothelial heparanase secretion, which in turn cleaves heparan sulfate, the main component of eGC (34). Increased heparanase production by Ang-2 was shown to be mediated by the inhibition of endothelium-stabilizing receptor Tie2 signaling (34). Additionally, Tie2 tyrosine kinase inhibition by Ang2 in sepsis resulted in a procoagulant phenotype of endothelial cells (44). Interestingly, increased Ang-2 has been associated with higher mortality, respiratory, liver, and kidney failure, and predicted DIC severity in patients with sepsis (44–47).

### Dysregulation of nitric oxide pathway

Nitric oxide (NO) is a soluble gas produced by endothelial cells and studied in depth for its protective role on the vascular endothelium. NO promotes vasodilation and is capable of inhibiting platelet activation and adherence to the endothelium (24). NO has another crucial role in impeding leucocyte adhesion on the vascular wall (24). This is possibly mediated through the downregulation of P-selectin, chemokine, E-selectin, ICAM-1, and VCAM-1 expression in endothelial cells (24). In preclinical sepsis models, an imbalance between endothelial nitric oxide synthase (eNOS) and inducible nitric oxide synthase (iNOS) activity has been demonstrated. Specifically, eNOS activity was found to be decreased compared to the upregulated iNOS activity. Overproduced NO by iNOS reacts with superoxide anion resulting in reactive nitrogen species (RNS) production, which contributes to endothelial dysfunction (38) (**Figure 1C**). Experimental and clinical studies are inconclusive regarding NO levels and their effects on the endothelium during sepsis. Some studies suggest that NO production increases during sepsis and this is associated with poor prognosis, while others support that NO levels are lower in sepsis patients compared to healthy controls (48–51). New evidence suggests that increased NO bioavailability in sepsis may be beneficial for the host without deteriorating vasodilation and subsequent hypotension, as has been proposed in previous studies (52). Overall, the dysregulation of the NO pathway during sepsis merits further investigation.

### Proadhesive endothelial cell phenotype

Expression of proadhesive molecules in activated endothelial cells poses a hallmark process for the progression of sepsis-induced endothelial dysfunction (**Figure 1D**). Activated endothelium interacts both with leukocytes and platelets, while platelet–leucocyte aggregates deposit on endothelium and further enhance local inflammation (23). The infection triggers immune and endothelial cells to produce reactive oxygen species (ROS) and inflammatory and anti-inflammatory cytokines. Reversely, cytokines stimulate endothelial cells to express adhesion molecules such as selectins, integrins, ICAMs, and VCAM-1 (38). These molecules interact with leukocytes, which roll, bind to the endothelium, and transmigrate through it. Furthermore, ROS released by immune cells during sepsis destroy the endothelium and result in increased permeability (38).

### Prothrombotic endothelial cell phenotype

Accumulating evidence suggests that endothelial cells can interact with immune cells, triggering thrombus formation, a process implicated in the pathogenesis of numerous diseases, such as antiphospholipid syndrome, coronary artery disease, and sepsis (53–58). Both experimental and clinical studies, presented in **Table 1**, explain how endothelial cells can lose their anticoagulant properties and lead to thrombosis when stimulated directly or indirectly by pathogens (**Figure 1E**). A large body of evidence suggests that upon sepsis-induced endothelium activation, there is increased TF expression and secretion accompanied by enhanced TF activity (26, 60, 62–64, 72). Complement, especially C5a, and the inactive terminal C complex (iTCC) formed by mixing C5b6, C7, C8, and C9, were also found to stimulate endothelial cells to produce active TF (77, 78) (**Table 2**). Beyond TF, other important mechanisms contributing to immunothrombosis are the dysfunctional fibrinolytic and anticoagulant properties of the endothelium. Accumulating experimental data confirm that pathogen invasion is associated with increased endothelial expression of PAI-1 and decreased expression of EPCR, TM, and TFPI, as well as decreased protein C activation (25, 63, 68, 69, 72). Complement C3 activation stimulated by Shiga toxin was also shown to result in endothelial TM reduction and enhanced thrombus formation (82) (**Table 2**). Despite decreased endothelial TM expression during sepsis, soluble TM is increased in patients with sepsis and predicts acute kidney injury, multiple organ failure, and mortality (96–99). A possible illustrative mechanism is that apoptotic endothelial cells release their intracellular proteins into circulation. Similarly, elevated TFPI plasma levels are associated with overt DIC in patients with sepsis, in contrast to the reduced TFPI expression by endothelium (100). Accordingly, increased plasma levels of PAI-1 have been correlated with DIC in patients with sepsis and predicted mortality (101). Circulating levels of proteins C and S have also been evaluated, and clinical data support their deficiency in sepsis (102). Importantly, in patients with severe sepsis, decreased plasma levels of protein C and increased syndecan-1, which characterize endothelial damage, were associated with whole blood hypocoaguability in the context of DIC, as measured by thromboelastography (103).

**Table 1:** Role of endothelial cells in immunothrombosis.

Population/cell type	Immune trigger	Prothrombotic endothelial response	Method of thrombosis assessment	Effect on thrombosis	Ref
In vitro					
HUVECs	TNF-α	↑ TF expression ↑ ICAM-1 (dependent on NF-κB)	N/A	N/A	(59)
HAECs	COVID-19 PRP-induced NETs	↑ TF expression ↑ ICAM-1 ↑VCAM-1	TAT complex levels measured by ELISA in the supernatant	↑ TAT complex	(60)
			TAT assay in cell culture supernatants	↑ TAT activity	
HUVECs	LPS or IL-6 or TNF-α or IL-1β	↑ PAI-1 production and release	N/A	N/A	(25)
HAECs	TNF-α	↑ PAI-1 mRNA dependent on sirtuin-1	N/A	N/A	(61)
Human saphenous vein ECs	PMA-induced NETs	↑ TF mRNA dependent on IL-1α and cathepsin G	TF activity measured as FXa generation by a chromogenic assay	↑ TF activity	(62)
			Plasma clotting measured by modified recalcification assay	Accelerated clotting	
			TF effect on coagulation capacity of human whole blood and plasma assessed by ROTEM	↓ TF-dependent coagulation time of whole blood and plasma	
HUVECs; HCAECs	IL-33	↑ total cellular TF protein ↑ TF cell surface levels ↑ TF mRNA dependent on IL-33 receptor and NF-κB ↓ TFPI protein levels and mRNA	TF activity measured as FXa generation by a chromogenic assay	↑ cell surface TF activity in ECs and in EC-derived microparticles	(63)
HUVECs	TNF-α	↑ TF expression in ECs and their microparticles	TF activity measured as FXa generation by a chromogenic assay	↑ TF activity	(64)
HUVECs	TNF-α; IL-6	↑ levels of full-length TF and its soluble isoform (asHTF) in cells and supernatants ↓ TFPI protein in cells and ↑ in supernatants	TF activity in HUVECs, cell culture supernatant, and cell-derived microparticles measured as FXa generation by a chromogenic assay	↑ TF activity in HUVECs (TNF-α>IL-6), ↑ asHTF and TF-bearing microparticle activity in the supernatant (only in TNF-α stimulation, not IL-6)	(26)
HGECs; HUVECs	TNF-α or combined TNF-α with Shiga toxin	↑ TF antigen and mRNA in HGECs (not measured in HUVECs) after TNF-α No additive effect of Shiga toxin	Cell surface TF activity measured as FXa generation by a chromogenic assay	↑ cell surface TF activity on HGECs and HUVECs (after TNF-α) TNF-α combined with Shiga toxin ↑ further cell surface TF activity on HGECs	(65)
HUVECs; HMVECs; HPAECs	LPS or TNF-α	↑ PAI-1 and PAI-2 mRNA and antigen (Ag) ↓ t-PA mRNA and antigen (Ag)	N/A	N/A	(66)
HUVECs; HCVECs	LPS (*E. coli* O111:B4)	↑ TF mRNA	TF activity measured by one-stage clotting assay	↑ TF activity	(67)
HUVECs; BAECs	HSV-1	↓ TM cell surface levels ↓ TM mRNA ↑ TF synthesis	TM activity measured by thrombin-dependent protein C activation	↓ surface TM activity	(68)
			TF activity measured by a modified one-stage clotting assay	↑ TF activity	
ECs from human umbilical artery and vein and from human foreskin microvessels	Human recombinant TNF-α or IL-1α or IL-1β or bacterial LPS	↑ PAI production	PAI activity in the supernatant fluid was assayed in the titration of t-PA in a fixed volume of conditioned medium	↑ PAI activity	(69)
Human ECs, isolated from neonatal umbilical cord veins, adult saphenous veins, adult iliac arteries, or adult thoracic aortae	Human recombinant TNF, IL-1	N/A	Total cellular procoagulant activity (PCA) measured by a standard one-stage clotting assay	↑ PCA (induced by TNF-α or IL-1 alone or in combination)	(70)
Ex vivo					
*A*orta segments from baboon	Lethal dose of *E. coli*	↑ TF expression on aortic ECs and on PSGL-1-bearing leukocytes in endothelium and subendothelium ↑ TF mRNA in the vascular wall	TF activity measured as FXa generation by a chromogenic assay	↑ TF activity (greater ↑ at branches)	(71)
			Immunofluorescence and confocal microscopy on aorta segments	Active ongoing coagulation process indicated by colocalization of TF with TAT complexes, FVIIa, FXa, activated PLTs, and fibrin in the arterial wall	
In vivo					
C57BL/6J mice	LPS (*Salmonella typhosa*)	↑ TF expression in the endothelial layer of aortic tissue	N/A	N/A	(59)
C57BL/6J mice	*E. coli* O111:B4	↓ phosphorylated (active) Tie2 ↓ Ang-1 (Tie2 agonist) ↑ Ang-2 (Tie2 antagonist) ↑ vWF, PAI-1, sE-selectin, sVCAM-1 (endothelial activation markers)	Plasma levels of D-dimers, PT, aPTT (coagulation assays)	↑ d-dimer, PT, aPTT	(44)
			Plasma levels of TAT complexes by ELISA	↑ TAT complexes dependent on the Ang-Tie2 axis	
FVB mice	*E. coli* LPS	Endothelial NF-κB activation ↑ TF expression on ECs in kidney tissue and isolated ECs from mice (dependent on NF-κB) ↓ EPCR and ↓ TM in heart, kidney, and liver tissue (dependent on NF-κB) ↑ plasma levels of PAI-1 (dependent on NF-κB)	Fibrinogen, d-dimers plasma levels	↓ fibrinogen, ↑ d-dimers	(72)
			Fibrin deposition in heart, liver, and kidney tissue sections by immunohistochemistry	↑ fibrin deposition in tissue sections of the heart, liver, and kidney (dependent on NF-κB activation)	
			APC plasma levels	↓ APC (dependent on endothelial NF-κB)	
TV616/TV614 mouse	LPS	Endothelial NF-κB activation	TAT complex plasma levels	↑ TAT complex, dependent on endothelial NF-κB activation	(73)
Rats	Zymosan A (fungi cell wall component)	↓ TM expression from ECs in alveolar capillaries	N/A	N/A	(74)
Baboon	Lethal dose of *E. coli*	↑ TF expression in ECs of the splenic microvasculature	PLTs, fibrinogen, and FDP blood levels	↓ PLT counts ↓ fibrinogen, ↑ FDP	(75)
			Immunohistochemistry in the splenic microvasculature	Presence of fibrin thrombi	
Clinical study					
Children with meningococcal sepsis and biopsy specimens of purpuric lesions from these patients	*Neisseria* meningitis	↓ expression of endothelial TM and EPCR both in vessels with and without thrombosis in children with sepsis vs. control	Assessment of thrombosis degree in each section of the skin-biopsy specimens	Presence of vessels with thrombosis in skin biopsy	(76)
			TAT complex, protein C Ag, protein S Ag, and antithrombin Ag plasma levels	↑ levels of TAT complexes and ↓ protein C, S, and antithrombin Ag plasma levels in sepsis children vs. control	

Ag, antigen; Ang, angiopoietin; APTT, activated partial thromboplastin time; APC, activated protein C; asHTF, alternatively spliced human TF; BAECs, bovine aortic endothelial cells; E. coli, Escherichia coli; ECs, endothelial cells; EPCR, endothelial protein C receptor; FDP, fibrin degradation products; FXa, factor Xa; HAECs, human aortic endothelial cells; HCAECs, human coronary artery ECs; HCVECs, human cardiac valve endothelial cells; HGECs, human glomerular endothelial cells; HSV-1, herpes simplex virus-1; HMVECs, human pulmonary microvascular endothelial cells; HPAECs, human primary pulmonary artery cells; HUVECs, human umbilical vein endothelial cells; ICAM-1, intercellular adhesion molecule 1; IL-6, interleukin-6; LPS, lipopolysaccharide; NETs, neutrophil extracellular traps; NF-κB, nuclear factor kappa B; PAI-1, plasminogen activator inhibitor-1; PLT, platelets; PMA, phorbol myristate acetate; PRP, platelet-rich plasma; PT, prothrombin time; ROTEG, rotational thromboelastography; TAT, thrombin–antithrombin; TF, tissue factor; TFPI, tissue factor pathway inhibitor; TM, thrombomodulin; TNF, tumor necrosis factor; t-PA, tissue-type plasminogen activator; u-PA, urokinase-type plasminogen activator; VCAM-1, vascular cell adhesion molecule 1. N/A, non applicable. ↑ (arrow pointing up) means increased. ↓ (arrow pointing down) means decreased.

**Table 2:** Complement role in immunothrombosis.

Study type	Complement activation trigger	Complement component	Thrombosis assessment method	Complement effect on thrombosis	Ref
In vitro					
Healthy mouse neutrophils	N/A	C5a	Quantification of NETosis *in vitro* with Sytox Green images of neutrophils	NETosis induction	(79)
Healthy human neutrophils	Serum from COVID-19 patients	C3a	TF mRNA levels (RT-PCR) TF protein detection by anti-TF mAb	↑ TF production and release	(60)
Healthy human neutrophils	Serum from COVID-19 patients	C5a	TF mRNA levels (RT-PCR) TF protein detection by anti-TF mAb	↑ TF production and release	(60)
			TF activity assessed by TAT assay	↑ TF activity	
			NET detection by ELISA of MPO/DNA complex levels and immunostaining	↑ NET production	
Mouse bone marrow PMNs	N/A	Recombinant mouse C5a	NETs detection by fluorescence microscopy	NETosis induction	(80)
Healthy human neutrophils	ANCA-associated vasculitis	C5a	TF expression on MPs and on NETs measured by anti-TF mAb	↑ release of TF-bearing MPs and TF-expressing NETs by neutrophils	(81)
			TAT-complexes in supernatants measured by ELISA	↑ TAT complexes dependent on TF from microparticles and NETs	
HMEC-1	Shiga toxin (*E. coli* 0157:H7)	C3a	Platelet adhesion assay under flow condition	Thrombi formation on the endothelial cell monolayer	(82)
			Anti-human TM followed by Cy3-conjugated secondary Ab	↓ TM expression on HMEC-1 surface	
Healthy human neutrophils	APS IgG	C5a	TF mRNA levels (RT-PCR)	↑ TF mRNA	(83)
			TF-mediated coagulation activity measured by mPT assay	↑ TF procoagulant activity	
Human mast cells (HMC-1) and basophils (KU 812)	N/A	Recombinant human C5a	PAI-1 Ag by ELISA using mAb	↑ PAI-1 Ag	(84)
			Active PAI-1 in conditioned media by specific, modified ELISA	↑ active PAI-1	
			PAI-1 mRNA (Northern blot) in HMC-1 cells	↑ PAI-1 mRNA	
HUVECs	N/A	Recombinant human C5a	TF mRNA levels (RT-PCR)	↑ TF mRNA	(85)
HUVECs	N/A	Inactive terminal C complex (iTCC) prepared by mixing C5b6, C7, C8, and C9	TF activity measured as FXa generation by a chromogenic assay	↑ endothelial TF activity	(78)
Human platelets	N/A	C1q coated surfaces Fluid-phase C1q multimers	Platelet aggregation monitored in a dual-channel aggregometer	↑ platelet aggregation	(86)
			Fibrinogen binding on platelets assessed in the presence of ^125^l-fibrinogen	↑ fibrinogen binding on GPIIb-IIIa complex	
			Platelets labeled with ^14^C-serotonin	↑ serotonin release	
			P-selectin (CD62) expression assessed by labeled antiCD62 mAb	↑ P-selectin expression	
			Surface procoagulant activity assessed by kaolin recalcification time of pooled normal plasma	↑ surface membrane procoagulant activity	
Healthy human PRP	N/A	Purified human C5b-9 complex	Dense granule secretion measured by the release of ^14^C serotonin and α-granule secretion monitored by the surface expression of GMP-140, detected by the radiolabeled mAb S12	↑ secretion of α- and dense granules	(87)
Healthy human PRP	N/A	C5b-9 complex bound on platelet membrane	Platelet prothrombinase activity assessed by thrombin generation in the presence of FVa and FXa	↑ rate of thrombin generation *via* platelet prothrombinase	(88)
			PF4 radioimmunoassay	↑ release of PF4	
Gel-filtered human platelets	N/A	C3a	Platelet aggregation measured by LTA	↑ platelet aggregation	(89)
			Platelets labeled with ^14^C-serotonin	↑ serotonin release	
Healthy human leukocytes	Contact of human plasma with renal dialysis membranes	Complement’s C5-derived fragments	TF activity measured by a two-stage assay	↑ TF activity	(90)
In vivo					
C57BL/6 mice	N/A	C5a	Histology and immunofluorescence staining of thrombi in arteries for NET detection	↑ areas of thrombus in mouse arteries ↑ NET formation in thrombi	(79)
Baboon	*E. coli* (sepsis model)	C5a	APTT, PT in plasma	↑ APTT and PT	(91)
			FXII, FXIa-AT, and FVIIa-AT complexes in plasma	↑ consumption of FXII, ↑ FXIa-AT, and FVIIa-AT complexes	
			TF mRNA levels	↑ TF mRNA	
			TAT complex, t-PA, PAI-1, plasmin-antiplasmin (PAP) complexes, d-dimer, FDP plasma levels	↑ TAT complexes, t-PA, PAI-1, PAP complexes, d-dimers, FDP	
Mice	Extracellular histones injection	C5a	Platelet blood count	↑ thrombocytopenia	(92)
			PT and APTT in plasma	↑ PT and APTT	
			Platelet aggregation measured by LTA	↑ platelet aggregation	
			Liver embolism detected by ↓ liver blood flow with ICG	Presence of liver embolism	
Mice C57BL/6	LPS and Shiga toxin (*E. coli* O157:H7)	C3a	Platelet blood count	↑ thrombocytopenia	(82)
			Fibrin(ogen) deposition in renal tissue determined anti-rat fibrin(ogen) Ab	↑ fibrin(ogen) deposition in glomerular capillary walls	
			Glomerular platelet clumps assessed by transmission electron microscopy	Presence of platelet clumps	
			TM detected by anti-TM Ab	↓ glomerular TM expression	
Baboon	*E. coli* (sepsis model)	C3a	Blood platelet count	↑ thrombocytopenia	(93)
			Immunolabeling: gpIIIa cell marker for platelets	↑ accumulation of platelets in the lung and on the surface of large vessels	
			Fibrinogen and FDP plasma levels, APTT in plasma	↓ fibrinogen, ↑ FDP, APTT	
			Quantitative immunofluorescence analysis and mRNA levels of PAI-1, TF, TFPI, and TM in lung cryosections	↑ protein and mRNA levels of TF, PAI-1 ↓ protein and mRNA levels of endothelial TFPI, TM	
			Histologic analysis of lungs right after euthanasia	↑ microvascular thrombosis	
Rats	CLP sepsis model	C5a	PT, APTT in plasma	↑ APTT and PT	(94)
			FV and FVII, fibrinogen, AT, plasminogen, t-PA, PAI, TAT complex, and d-dimers levels in plasma	↓ FVII:C, ↑ fibrinogen, ↓ AT, ↓ plasminogen, ↑ t-PA, ↑PAI, ↑ TAT complex, ↑ d-dimers	
			Platelet count in PRP	↑ thrombocytopenia	
Clinical study					
Prospective clinical trial, two patients with hemolytic PNH and a control group (*n* = 23)	Hemolytic PNH-induced complement injury	CD55 and CD59	d-dimers, plasma TAT complex levels by ELISA	↑ d-dimers and TAT complexes	(95)
			Leucocyte-derived TF plasma levels using anti-CD162 mAb	↑ TF expression derived from monocytes and macrophages	

AT, antithrombin; APS, antiphospholipid syndrome; C5aR, C5a receptor; CLP, cecal ligation and puncture; ELISA, enzyme-linked immunosorbent assay; FDP, fibrinogen degradation products; Fib, fibrinogen; FXa, factor Xa; GMP, granule membrane glycoprotein; HMEC-1, human microvascular endothelial cell line of dermal origin; HUVEC, human umbilical endothelial cell; ICG, indocyanine green; LTA, light transmission aggregometry; mAb, monoclonal antibody; mPT, modified prothrombin time; MP, microparticle; MPO, myeloperoxidase; NETs, neutrophil extracellular traps; PF4, platelet factor 4; PLA, platelet-leukocyte aggregate; PMNs, polymorphonuclear leukocytes; PNH, paroxysmal nocturnal hemoglobinuria; PRP, platelet rich plasma; TAT, thrombin–anti-thrombin; TF, tissue factor; t-PA, tissue plasminogen activator. N/A, non applicable. ↑ (arrow pointing up) means increased. ↓ (arrow pointing down) means decreased.

### Transcription factor activation

Endothelial cell activation, following pathogen invasion, is mediated by intracellular signaling, which includes transcription factor activation such as nuclear factor kappa B (NFκB), activator protein-1 (AP-1), interferon regulatory transcription factor-3 (IRF3), and signal transducer and activator of transcription 3 (STAT3) (38, 104) (**Figure 1A**). Specifically, activation of endothelial NFκB by pathogens has been found to mediate increased expression of procoagulant molecules such as PAI-1 and TF, while fibrinolytic and anticoagulant factors such as TM, APC, and EPCR diminished in a way that was also dependent on NFκB (72, 73). Indeed, NFκB has been associated with increased circulating d-dimers and tissue fibrin deposition in an endotoxemia mouse model (72).

### Role of Weibel–Palade bodies

Weibel–Palade bodies (WPBs) are organelles inside endothelial cells containing procoagulant proteins such as vWF, P-selectin, and factor VIII (FVIII), which can rapidly undergo exocytosis under specific circumstances (33). Lipopolysaccharide (LPS) has been shown to induce WPBs’ content secretion both *in vitro* and *in vivo* (33) (**Figure 1E**). Ultra-long vWF strings can be produced *in vitro* after endothelial cell activation by Shiga toxin. Furthermore, the Shiga toxin prevented cleavage of these vWF strings by inactivating ADAMTS13 (105). It has been found that in preclinical sepsis models, increased markers of endothelial activation are associated with abnormal coagulation markers, while some studies have additionally demonstrated the subsequent thrombus formation in the microcirculation. Dysregulated coagulation was indicated by markers such as high levels of circulating d-dimers, fibrin, fibrin degradation products (FDPs), thrombin-antithrombin (TAT) complexes prolonged prothrombin time (PT), activated partial thromboplastin time (aPTT), and/or decreased levels of plasma fibrinogen and platelets (44, 71, 72).

## Neutrophils in immunothrombosis

**Figure 2:**
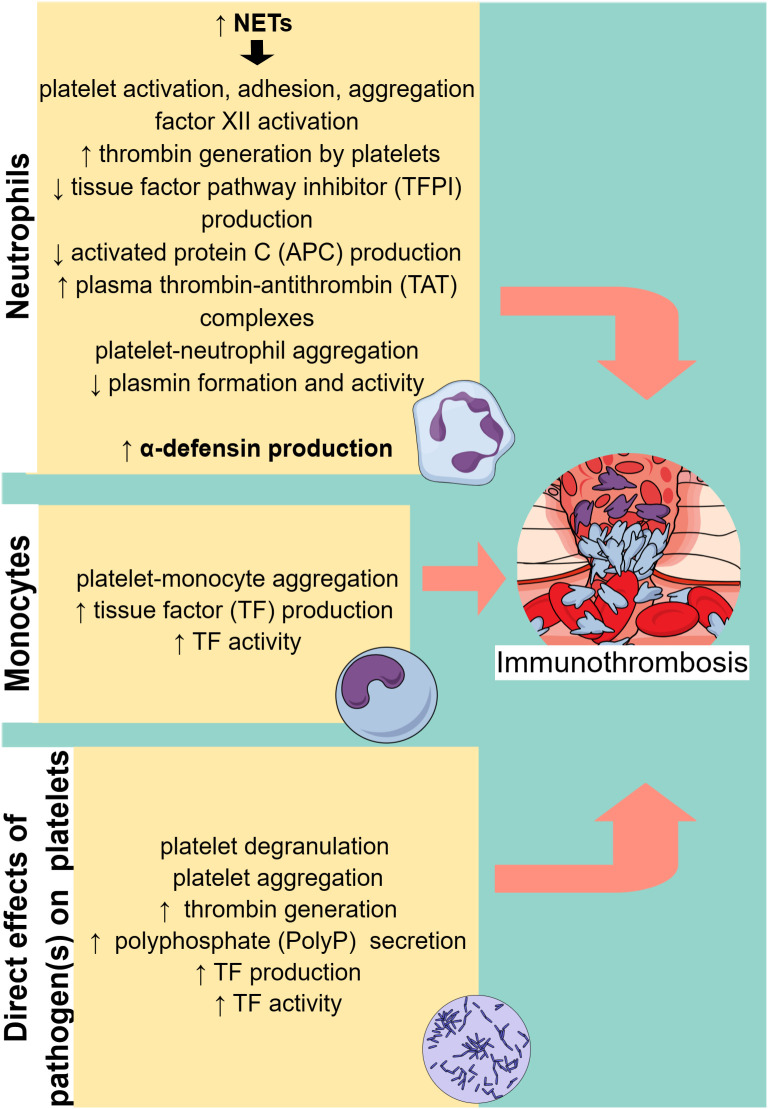
Cell-specific immunothrombosis mechanisms. Neutrophils and monocytes can induce thrombosis through specific mechanisms, while pathogens can directly activate platelets, leading to thrombosis. NETs, neutrophil extracellular traps. ↑ (arrow pointing up) means increased. ↓ (arrow pointing down) means decreased.

Apart from endothelial dysfunction, which facilitates immunothrombosis in sepsis, activated cells of the innate immune system, in particular neutrophils and monocytes, also play a significant role (**Figure 2**). Neutrophils are an essential part of the host’s innate immune response and are typically the first responders to acute inflammation (28). In sepsis, PAMPs and damage-associated molecular patterns (DAMPs) can activate neutrophils by binding to PRRs. Activated neutrophils exert their antimicrobial activity mainly through three processes: phagocytosis, degranulation, and the release of NETs (28).

### Induction of NETosis in sepsis

A variety of different pathogens, bacterial toxins, and cytokines can induce NETosis (28, 106). NETosis can also be enhanced by activated platelets (28, 60, 107–109). More specifically, in a sepsis mouse model, it was found that LPS binding to platelet TLR4 stimulated platelets to interact with neutrophils and induce NET production in the liver sinusoids and capillaries (107). The binding of platelet P-selectin to P-selectin glycoprotein ligand 1 (PGSL1) on neutrophils was found to be a key mechanism for NETosis induction in mice (110). However, this finding was not reproduced in studies with human neutrophils, and different agonists, where an antibody against P-selectin did not affect platelet-mediated NETosis (111, 112). The interaction of platelet-derived high mobility group protein B1 (HMGB1) with the receptor for advanced glycation end products (RAGE) on neutrophils was also found to be a potential mechanism for NETosis stimulation by platelets, possibly through induction of autophagy in neutrophils (112, 113). Moreover, it was shown that LPS and Pam3CSK4, a synthetic lipopeptide, through binding to platelet TLR4 and TLR2, respectively, activated platelets to interact with neutrophils and induce NETosis through platelet GP1b-neutrophil β2-integrin (CD18) interaction. Platelet-derived vWF, platelet factor 4 (PF4), and thromboxane A2 (TXA2) were essential mediators of this process (111). Furthermore, experimental studies have shown that platelet-derived CXCL4/CCL5 heterodimers and β-defensin 1 enhanced NET production (114). Besides platelet–neutrophil interaction and subsequent NETosis induction, it was found that also complement cleavage products, in particular, C3a and C5a anaphylatoxins could stimulate NETosis in sepsis animal models or in neutrophils stimulated by LPS or platelet-rich plasma from COVID-19 patients (60, 80, 115–117) (**Table 2**). Moreover, complement may play a role in the inhibition of NET degradation (118).

### Role of NETs in endothelial dysfunction

It has been shown that NETs, apart from their beneficial role in clearing the invading microorganism, are implicated in the pathogenesis of many diseases in which endothelial dysfunction is present, such as sepsis and cardiovascular diseases. Therefore, there has been a growing interest in studying the role of NETs in endothelial dysfunction in sepsis (119). *In vitro* studies suggest that NETs can promote endothelial dysfunction, as indicated by increased endothelial expression of ICAM-1, VCAM-1, E-selectin, TF, and vWF (60, 62, 109, 120–123). Cathepsin G is a component of NETs, capable of processing pro-IL-1α into IL-1α, which has been found to enhance VCAM-1 and ICAM-1 expression on the endothelial surface (62). Moreover, NET-induced ICAM-1 and E-selectin expression on endothelial cells was found to be dependent on the triggering receptor expressed on myeloid cell-1 (TREM-1). TREM-1 is an orphan immune receptor expressed on various types of cells, such as immune cells, platelets, and endothelial cells. Apart from mediating the NET-induced proadhesive phenotype of endothelial cells, TREM-1 potentiated NETosis triggered by LPS (121). NETs have also been associated with increased platelet adhesion on endothelial cells through enhancement of vWF expression on the endothelium (120). Both NETs and histones have been found to increase TF mRNA and TF activity in human aortic endothelial cells (HAECs) and human saphenous vein endothelial cells (HSVECs), resulting in plasma clotting acceleration (60, 62, 123). Specifically for histones, their effect on TF was mediated by TLR2 or TLR4, which in turn activated AP-1 and NFκB transcription factors (123). As one of the major components of NETs, histones have been further studied for their impact on endothelium, with results suggesting cytotoxicity in a concentration-dependent manner (124). Citrullinated histone H3 (CitH3) was found to increase endothelium permeability *in vitro* (125). Furthermore, images using intravital microscopy showed that CitH3 resulted in microvascular endothelial barrier dysfunction, by disrupting the adherens junctions and reorganizing the actin cytoskeleton without causing cell death (126). Accordingly, NETs have been found to decrease endothelial expression of VE-cadherin and Zonula occludens (ZO)-1 proteins, which are major components of adherens and tight junctions, respectively (109).

### Prothrombotic role of NETs

NETs not only contribute to endothelial dysfunction but have also been found to exert procoagulant and prothrombotic activity through various mechanisms, as shown in **Table 3**. NETs could serve as a scaffold and inducer of platelet adhesion, activation, and aggregation, and this effect was largely mediated by specific NET components like histones H3 and H4 (134). Nets not only served as a scaffold for platelet adhesion but also for red blood cells (RBCs), promoting the generation of red (RBC-rich) thrombus. Plasma proteins, implicated in thrombosis, such as vWF, fibronectin, and fibrinogen could also bind to NETs, while NETs contributed to thrombin-mediated fibrin generation (134). Histones, in particular H3 and H4, could activate platelets to promote plasma thrombin generation, through binding to platelet TLR2 and TLR4 (131). Notably, this effect was enhanced when histones were in complex with DNA (131). In contrast, another study showed that the addition of deoxyribonuclease (DNase), which destroys the DNA network of NETs, enhanced histone-mediated, platelet-dependent thrombin generation, presumably due to increased exposure to histones (128). Besides NETs-induced platelet-dependent mechanisms of thrombin formation, NETs were capable of stimulating thrombin generation also in platelet-poor plasma *via* activation of the intrinsic coagulation pathway (128). This effect was largely attenuated with DNase administration, suggesting the crucial role of cell-free DNA (cfDNA) in that process. Accordingly, DNA–histone complexes measured in platelet poor plasma from sepsis patients were increased compared to plasma from healthy subjects, and cfDNA was positively correlated with thrombin generation (128). NETs, possibly due to their negatively charged extracellular DNA surface, could bind and promote activation of factor XII, thus triggering the contact pathway of coagulation (146). Interaction of NETs with membrane-derived microparticles released by activated neutrophils enhanced NET-mediated intrinsic coagulation pathway activation and subsequent thrombin formation (139). In support, it was demonstrated that NETs were capable of promoting pulmonary clot formation in mice with septicemia caused by LPS and heat-killed *E. coli* (140). However, despite robust evidence on the effect of NET components on thrombosis, the role of intact NETs may merit further investigation. Specifically, an *in vitro* study showed that although cfDNA and histones, separately, were capable of coagulation activation, intact NETs could not (147). One possible explanation for this is that the complex interactions between DNA and histones within the NET structure abrogate the thrombogenic negative charge of DNA and cover the histones’ binding sites. Moreover, the absence of other blood cells, endothelium, and blood flow in an *in vitro* model compared to an *in vivo* one may have contributed to the absence of the procoagulant effect of intact NETs (147).

**Table 3:** Role of neutrophils in immunothrombosis.

Population/cell type	Immune trigger	Prothrombotic neutrophil response	Method of thrombosis assessment	Effect on thrombosis	Ref
In vitro					
Healthy human neutrophils	PMA-induced NETs	NETs inactivate the plasma natural anticoagulant ZPI	Time course of FXIa and FXa inhibition when incubated with NET-bound ZPI or ZPI alone using a continuous chromogenic assay	Inability of FXIa and FXa inhibition by NET-bound ZPI	(127)
Platelet-rich plasma	PMA-induced NETs	N/A	Thrombin generation monitored using the Technothrombin TGA thrombin generation assay	↓ lag time and peak time to thrombin generation ↑ peak and total thrombin generation (mediated by histones and platelet TLR-2 and TLR4)	(128)
Platelet-poor plasma	PMA-induced NETs	N/A	Thrombin generation monitored using the Technothrombin TGA thrombin generation assay	↑ thrombin generation (dependent on cfDNA and intrinsic coagulation pathway activation)	(128)
Fibrin-NET matrices	DNA-bound elastase of PMA-induced NETs	N/A	Identification of plasminogen fragments by Western blot Conversion of plasminogen into plasmin assessed by a plasmin-selective chromogenic substrate	↑ plasminogen degradation, ↓ plasmin formation	(129)
Human PMNs	Thrombin-activated platelets in plasma from patients with sepsis-induced ARDS	↑ TF protein plasma levels and mRNA ↑ TF-enriched NETs	TAT complex levels measured by ELISA	↑ plasma TAT complex levels	(109)
Healthy human neutrophils	PRP from COVID-19 patients	↑ TF mRNA ↑ TF-bearing NETs, identified by MPO/DNA complexes	TAT complex levels measured by ELISA	↑ TAT complex levels in supernatant dependent on NETs	(60)
Healthy human neutrophils	Plasma or platelets from patients with sepsis	↑ NET production	Thrombin generation assessed by measuring TAT complex levels Fibrin formation monitored by measuring the optical density (405 nm) of the plasma on a Spectramax microplate reader	↑ thrombin and fibrin generation	(130)
Human platelet-rich plasma	Recombinant histones H3 and H4 (NET component)	N/A	Plasma thrombin generation analyzed by the CAT method	↑ thrombin generation (platelet TLR-2 and TLR-4 dependent)	(131)
Gel-filtered human platelets	Recombinant histones H3 and H4 (NET component)	N/A	Platelet aggregation optically monitored in a four-chamber aggregometer	↑ platelet aggregation	(131)
			Prothrombinase activity measured by thrombin generation	↑ platelet prothrombinase activity	
			P-selectin and Factor V/Va exposure on platelet surface assessed by flow cytometry	↑ P-selectin and Factor V/Va (platelet TLR-2 and TLR-4 dependent)	
Platelet-poor plasma	Human recombinant histones H3 and H4 (NET component)	N/A	Plasma thrombin generation analyzed by the CAT method	↑ plasma thrombin generation	(132)
			TM-mediated protein C activation assay	↓ TM-mediated protein C activation	
Plasma from healthy controls (*n* = 10)	Exogenous cfDNA	N/A	Lysis times determined as the time to half maximal ↓ in absorbance monitored at 405 nm in a SpectraMax M5e plate reader	↑ clot lysis time	(133)
			Plasminogen activation assay, binding interaction assessed by surface Plasmon resonance, effects of cfDNA on the plasmin-fibrin interaction assessed by a fluorometric displacement assay	↓ plasmin activity (*via* the formation of DNA/plasmin/fibrin complex	
Plasma from patients with sepsis	↑ cfDNA	N/A	Lysis times determined as the time to half maximal ↓ in absorbance (defined as complete clot lysis) monitored at 405 nm in a SpectraMax M5e plate reader	Delayed and ↓ clot lysis	(133)
			d-dimers quantified by immunoassay	↓ d-dimers (↓ fibrin degradation)	
Plasma from patients with sepsis	↑ cfDNA	N/A	Thrombin generation monitored using the Technothrombin TGA thrombin generation assay	↑ thrombin generation	(128)
Healthy human neutrophils	PMA-induced NETs	N/A	Platelet aggregation determined by an optical aggregation system after NET infusion on whole blood at high or low shear rates	↑ platelet aggregation on NETs	(134)
Healthy human neutrophils	BALF from ARDS patients	↑ TF expression dependent on C5a and TNF-α	TF-mediated coagulation activity measured by mPT assay	↑ TF procoagulant activity	(135)
In vivo					
C57BL/6 J mice	CLP sepsis model	↑ NET formation identified by MPO, CitH3	Microthrombus formation using H&E staining of liver sections	↑ microthrombus formation in central liver veins	(136)
C57BL/6J mice	CLP sepsis model	↑ TF-enriched NETs	NET detection in lung thrombi by immunofluorescence staining and immunoblotting for TF determination	↑ TF-enriched NETs in lung thrombi	(109)
C57BL/6J CD45.2 mice	LPS (*E. coli* 0111/B4)	↑ plasma NETs identified by cfDNA, NE, citH3 complex in miR-146a KO mice	Plasma TAT complex levels measured by ELISA	↑ TAT complex in miR-146a KO mice	(137)
C57Bl/6 mice	LPS (*E. coli* 0111:B4)	↑ NET release	Thrombin generation detected by cleavage of a quenched-fluorescence peptide thrombin substrate	↑ thrombin generation within the liver sinusoids	(138)
			Confocal intravital microscopy to visualize liver and lung microcirculation	↑ platelet aggregation and immunothrombi in liver and lung microcirculation	
C57BL/6 mice	CLP sepsis model	↑ NET- and membrane-derived MP formation ↑ NET-MP complexes	Thrombin formation monitored by measuring accumulated fluorescence using a microplate reader	↑ thrombin generation *via* intrinsic coagulation pathway activation	(139)
			Plasma TAT complex levels measured by ELISA	↑ TAT complexes	
C57BL/6 mice	Injection of a G-CSF-expression plasmid (Csf3)	↑ blood neutrophils ↑ NET-like structures in blood smear	Quantification of blood vessels in lungs occluded by hematoxylin-positive clots per field of view	↑ occluded blood vessels dependent on DNase inactivation	(140)
C57Bl/6J mice	LPS (*E. coli* 0111:B4)	↑ NET production	Quantitative analysis of thrombin activity by thrombin probe fluorescence	↑ intravascular thrombin activity dependent on histone H4	(108)
			SD-IVM images within the liver, lung, and mesentery microcirculation	↑ colocalization of intravascular NETs and dense thrombin activity in the microvasculature of the liver, lung, mesentery	
C57Bl/6J mice	LPS (*E. coli* 0111:B4), *S. aureus*	↑ NET production dependent on PAD4	Quantitative analysis of thrombin activity by thrombin probe fluorescence	↑ intravascular thrombin activity dependent on PAD4	(108)
SV129S1 mice	*E. coli*	↑ NETs identified by externalized nucleosomes	TFPI degradation assessed by Western blot (TFPI and TFPI degradation products) in mice thrombi	↑ TFPI degradation dependent on NETs	(141)
			Fibrin deposition in liver and spleen tissue sections visualized with specific antibody	Fibrin deposition inside vessels of the hepatic microvasculature, partial or total occlusion from fibrin clots (94% of occluded vessels were sinusoids)	
C57BL/6 mice	Histones (NET component)	N/A	H&E and Mallory’s phosphotungstic acid-hematoxylin staining for fibrin	Presence of platelet and fibrin-rich microthrombi in lungs	(142)
Clinical studies					
COVID-19 patients (*n* = 127) with control group (*n* = 15)	SARS-CoV-2 infection	↑ plasma neutrophil-released α-defensin in COVID-19 patients	Measurement of d-dimers peak plasma levels	↑ d-dimers in the COVID-19 group, correlated with α-defensin plasma levels	(143)
COVID-19 patients (*n* = 19) with control group (*n* = 13)	SARS-CoV-2 infection	↑ plasma neutrophil-released α-defensin and ↑ IL-6 in COVID-19 patients	Clot formation assayed by TEG	Acceleration of clot formation in the COVID-19 group	(143)
COVID-19 patients (*n* = 25) with control group (*n* = 10)	SARS-CoV-2 infection	↑ plasma NETs in the COVID-19 group ↑ neutrophil TF mRNA in the COVID-19 group Colocalization of TF and NETs on neutrophils of COVID-19 patients	Plasma TAT complex levels measured by ELISA	↑ plasma TAT complex levels in the COVID-19 group, correlated with ↑ NETs	(60)
Patients with septic shock and DIC (*n* = 10) and without DIC (*n* = 10)	Septic shock	↑ plasma NETs (DNA bound MPO), ↑ circulating nucleosomes	DIC diagnosis according to JAAM-DIC diagnostic criteria	DIC diagnosis	(144)
Patients with sepsis (*n* = 52) and nonsepsis (*n* = 10) in ICU, healthy controls (*n* = 40)	Sepsis	↑ circulating levels of cfDNA, NE, and MPO-DNA complex in sepsis patients	Plasma TAT complex and d-dimer levels	↑ plasma levels of TAT and d-dimers ↑ Caprini score for VTE	(130)
Healthy humans (*n* = 16)	LPS (*E. coli* O:113)	↑ plasma histone complexed DNA (hcDNA) ↑ neutrophil-platelet aggregates	Clotting time and clotting formation time assayed by ROTEM	↓ clotting time and clotting formation time	(145)
			Plasma prothrombin fragments 1 and 2 (F1+2)	↑ F1+2	
			Plasma TAT complex levels	↑ plasma TAT complexes	

ARDS, acute respiratory distress syndrome; BALF, bronchoalveolar lavage fluid; CAT, calibrated automated thrombinography; cfDNA, cell-free DNA; CitH3, citrullinated histone H3; CitH4, citrullinated histone H4; DIC, disseminated intravascular coagulation; E. coli, Escherichia coli; H&E, hematoxylin and eosin; JAAM, Japanese Association for Acute Medicine; LPS, lipopolysaccharide; MPO, myeloperoxidase; MPs, microparticles; mPT, modified prothrombin; NE, neutrophil elastase; NETs; neutrophil extracellular traps; PAD4, peptidyl arginine deiminase 4; PMA, phorbol 12-myristate 13-acetate; PMNs, polymorphonuclear neutrophils; PRP, platelet-rich plasma; ROTEM, rotational thromboelastometry; SARS-CoV-2, severe acute respiratory syndrome coronavirus 2; SD-IVM, spinning-disk confocal intravital microscopy; TAT, thrombin–antithrombin; TEG, thrombelastography; TLR, toll-like receptor; TF, tissue factor; TFPI, tissue factor pathway inhibitor; tPA, tissue plasminogen activator; VTE, venous thromboembolism; ZPI, Z-dependent protease inhibitor. N/A, non applicable. ↑ (arrow pointing up) means increased. ↓ (arrow pointing down) means decreased.

### Disruption of endogenous anticoagulant and fibrinolytic mechanisms by NETs

NETs were also shown to interfere with the endogenous anticoagulant mechanisms. More specifically, extracellular nucleosomes within NETs facilitated TFPI degradation by neutrophil elastase on the surface of activated neutrophils, thus abrogating TFPI-mediated inhibition of of the extrinsic coagulation pathway (141). Neutrophil elastase bound to DNA complexes was also shown to cleave plasminogen into fragments, resulting in decreased plasmin production and impaired fibrinolysis (129). Apart from decreased plasmin generation, cfDNA was capable of binding to plasmin and fibrin at the same time, and the formed complex was shown to be responsible for decreased plasmin-mediated fibrin clot dissolution (133). Besides cfDNA, histones, specifically H3 and H4, could also interact with TM and protein C, leading to the inhibition of APC generation (132).

### Prognostic utility of NETs in sepsis

Therefore, experimental data support the crucial role of NETs in triggering thrombus formation. From a clinical point of view, it was shown that increased NET-forming capacity was significantly associated with thrombocytopenia, increased PT, aPTT, d-dimers, and low fibrinogen in patients with sepsis at intensive care unit (ICU) admission, while it could also predict DIC development and mortality after ICU admission (106). Noteworthy, specific components of NETs, such as circulating levels of cfDNA, neutrophil elastase, and the myeloperoxidase–DNA complex in patients with sepsis, are positively correlated with the risk of VTE (130). Increased MPO-DNA levels on days 3 and 7 during sepsis hospitalization were associated with decreased mean arterial pressure, a lower PaO_2_/FIO_2_ ratio, an increased sepsis-related organ failure assessment (SOFA) score, and 28-day mortality (148).

### TF production and release by neutrophils

Regarding the production and release of TF by neutrophils, there is conflicting evidence. It was reported that neutrophils in the setting of appropriate interaction with injured endothelial cells or after stimulation with P-selectin or the peptide formyl-MetLeuPhe (Fmlp) were able to produce functional TF, initiating the extrinsic coagulation pathway (149, 150). Moreover, C3a and C5a anaphylatoxins were found to stimulate neutrophils to produce and release active TF (**Table 2**) (60, 83, 90). However, these findings have been questioned in other studies, and it was proposed that granulocytes may acquire TF produced by monocytes (151, 152). With respect to the presence of TF in NETs, it was found that neutrophils from patients with sepsis could release large amounts of functional TF-bearing NETs, and autophagy was implicated in that process (151).

### Role of neutrophil-derived α-defensins in immunothrombosis

Another potential mechanism through which neutrophils may contribute to immunothrombosis is by secreting antimicrobial peptides. Specifically, α-defensins, which are secreted by neutrophils as part of the host innate immune response, have been found elevated in the plasma of septic patients (153). Recently, an *in vivo* study showed that α-defensins accelerate and stabilize fibrin clot formation, while also disrupting fibrinolysis (154). Consistently, circulating α-defensins have been correlated with the acceleration of clot formation and d-dimers plasma levels in COVID-19 patients (143, 155).

## Monocytes in immunothrombosis

Monocytes and macrophages have been found to play a vital role in sepsis-induced immunothrombosis (**Figure 2**). TF expression by monocytes seems to be the most potent pathway leading to coagulation cascade activation in sepsis (156–160). While both hematopoietic and non-hematopoietic cells produce TF and enhance coagulation in an endotoxemia mouse model, monocytes are the main source of circulating TF (161). Additionally, other blood cells have been found to acquire TF released by monocytes through microparticles (161). Numerous studies depicted in **Table 4** suggest that pathogens induce monocyte-derived TF expression and release in the circulation, which in turn activates the extrinsic coagulation pathway. The binding of LPS to transmembrane receptors such as TLR4 in monocytes induces TF mRNA expression *via* NF-κB activation (166). Moreover, the interaction of pathogen components either with TLRs or directly with intracellular pathways in monocytes can result in inflammasome activation and subsequent TF release *via* pyroptosis (163, 166). Pyroptosis is a type of proinflammatory cell death induced by caspase-1 (canonical inflammasome) or caspase-11 (noncanonical inflammasome) and is characterized by gasdermin D-dependent pore formation in the cell membrane, subsequent osmotic cell lysis, and finally the release of cytosolic content. Indeed, LPS or type III secretion system (T3SS) rod proteins have been found to trigger *in vivo* caspase-11 and caspase-1 activation, respectively, leading to TF release (163). Gasdermin D-mediated pore formation induced by caspase-11 has also been proposed to activate TF on macrophages (162). Specifically, pore formation on the cell membrane can induce calcium influx, which triggers phosphatidylserine exposure on the membrane, followed by TF activation (162). Furthermore, sphingomyelin, another membrane lipid, is involved in the activation of TF to its procoagulant form. This process occurs on the macrophage cell membrane and is dependent on sphingomyelin hydrolysis by acid sphingomyelinase (ASMase) (167).

**Table 4:** Role of monocytes/macrophages in immunothrombosis.

Population/cell type	Immune trigger	Prothrombotic monocytes/macrophage response	Method of thrombosis assessment	Effect on thrombosis	Ref
In vitro					
C57BL/6J mice; macrophages	LPS delivered by cholera toxin subunit B (CTB)	↑ phosphatidylserine exposure on the cell membrane ↑ TF mRNA	TF activity assessed by a chromogenic assay Thrombin production assessed by fluorimetric assay	↑ TF activity and ↑ thrombin production are dependent on caspase 11, gasdermin D, and phosphatidylserine	(162)
			Fibrin formation assay	↑ fibrin production dependent on TF and phosphatidylserine	
THP-1 cells	EprJ (*E. coli* T3SS rod protein)	↑ TF release	TF activity measured as FXa generation by a chromogenic assay	↑ TF activity in the supernatant	(163)
Mouse primary bone marrow-derived macrophages	LPS (*E. coli* O111:B4)	↑ TF release, dependent on caspase 11	N/A	N/A	(163)
Healthy human PBMCs	LPS	↑ surface TF	Thrombin generation assay	↑ thrombin generation by TF-bearing PBMC dependent on PDI	(157)
Healthy human monocytes	LPS (*E. coli* 0127:B8)	↑ TF mRNA	TF activity measured as FXa generation by a chromogenic assay	↑ TF activity	(156)
Healthy human monocytes	LPS	↑ TF expression and generation of microparticles containing TF	N/A	N/A	(164)
Healthy human whole blood	LPS	↑ TF expression by monocytes and their microparticles	N/A	N/A	(164)
Healthy human monocytes	LPS (*E. coli* O111:B4)	↑ Microvesicle production contained increased TF and PSGL-1 Fusion of monocyte-derived microvesicles with activated platelets Platelets acquire PSGL-1 *via* P-selectin and TF	TF activity measured as FXa generation by a chromogenic assay	↑ TF activity in microvesicle-platelet supernatants	(158)
Healthy human monocytes	LPS	N/A	Assay for monocyte TF activity	↑ TF activity	(160)
THP-1 cells from a human monocytic cell line	TNF-α	↑ production of microparticles abundant in TF and CD15	TF activity measured as FXa generation by a chromogenic assay	↑ TF activity in microparticle suspension	(159)
In vivo					
Mice C57BL/6J	LPS (*E. coli* O111:B4)	N/A	PT determined with thromboplastin-D Plasma TAT concentrations determined using a mouse TAT ELISA kit Fibrinogen plasma concentration Platelet count	↑ PT, ↓ fibrinogen, ↑ TAT, ↓ and platelet count, all dependent on caspase-11 and gasdermin D	(163)
Mice C57BL/6J	EprJ (*E. coli* T3SS rod protein)	N/A	PT determined with thromboplastin-D	↑ PT and	(163)
			Plasma TAT concentrations determined using a mouse TAT ELISA kit	↑TAT dependent on TF, monocytes/macrophages, caspase-1, and gasdermin D	
			Fibrinogen plasma concentration	↓ fibrinogen dependent on monocytes/macrophages, caspase-1, and gasdermin D	
			TF activity measured as Xa generation by a chromogenic assay	↑ TF activity in plasma microvesicles (MVs) dependent on caspase-1 and gasdermin D	
			Fibrin deposition detected by immunostaining in the liver and spleen	↑ fibrin deposition	
			Platelet count	↓ platelet count	
Clinical study					
Prospective clinical trial, 13 healthy humans	LPS (*E. coli*)	↑ platelet-monocyte aggregates in whole blood ↑ TF on monocytes	N/A	N/A	(165)

CD40L, CD40 ligand; E. coli, Escherichia coli; FXa, activated factor X; LPS, lipopolysaccharide; PBMCs, peripheral blood mononuclear cells; PDI, protein disulfide isomerase; PNH, paroxysmal nocturnal hemoglobinuria; PT, prothrombin time; PSGL-1, P-selectin glycoprotein ligand-1; TAT, thrombin antithrombin; TF, tissue factor; TNF-α, tumor necrosis factor-a; T3SSs, type III secretion systems. N/A, non applicable. ↑ (arrow pointing up) means increased. ↓ (arrow pointing down) means decreased.

Tissue factor production is additionally triggered by HMGB1, which is a DAMP released by myeloid cells, platelets, and hepatocytes. HMGB1 binds LPS, enhances endocytosis of the LPS-HMGB1 complex in monocytes, and is associated with increased TF release *via* caspase-11-induced pyroptosis (168). Furthermore, TF expression and activity are also induced by the interaction of monocytes or their microparticles with activated platelets (158, 164). Monocyte activation by pathogens is followed by increased PSGL-1 expression and the release of TF- and PSGL-1-bearing microparticles. These microparticles can fuse *in vitro* with platelets, leading to increased TF activity (158). Additionally, a prospective clinical study confirms that LPS endotoxemia enhances platelet–monocyte aggregation, TF expression, and platelet activation (165). On the other hand, thrombin generated by coagulation cascade activation binds to monocytes *via* protease-activated receptor-1 (PAR-1), leading to increased TF expression. Accordingly, inhibition of PAR-1 by vorapaxar (a PAR-1 antagonist) has been found to attenuate sepsis-induced coagulation, as indicated by decreased plasma prothrombin fragments and TAT complexes (169).

## Platelets in immunothrombosis

### Platelet activation in sepsis

Accumulating evidence suggests a complex interaction between platelets and different immune cells. Increased platelet activation and platelet–leukocyte aggregates have been found in a variety of thrombotic conditions, such as unstable angina, myocardial infarction, stroke, and sepsis (170–173). Multiple roles of platelets have been demonstrated in experimental and clinical studies. Platelets were suggested to contribute to tissue regeneration and myocardial recovery following acute coronary syndrome (ACS) (174–178). On the contrary, increased platelet activation and reactivity have been linked with a poor prognosis in several diseases, including ACS (179), dementia (180), diabetes (181, 182), and sepsis (183). Specifically, in sepsis, thrombocytopenia is associated with increased disease severity and an ominous prognosis (184). Decreased platelet count is attributed to excessive peripheral consumption of platelets resulting from many causes, among which are the interaction of platelets with immune cells, the microcirculation thrombosis, and the direct binding of pathogens to platelets (184). Beyond innate immune cells, which play a crucial role in immunothrombosis, pathogens can directly activate platelets, which in turn promote thrombosis (**Figure 2**). Recently, it has been found that during sepsis there is accelerated megakaryocyte maturation, triggered by PAMPs, DAMPs, TNF-α, or other cytokines. This subpopulation of megakaryocytes presents a stronger immunological profile and produces fewer platelets compared to megakaryocytes undergoing normal maturation induced by thrombopoietin (185). A variety of receptors on platelets interact with PAMPs, or pathogens coated by antibodies. Numerous pathogens have been proven to activate platelets and increase their aggregation, as presented in **Table 5**. Pathogens coated by immunoglobulins or pentraxins bind to low-affinity receptors for the constant fragment (Fc) of immunoglobulin (Ig) G (FcγRIIa) on platelets, leading to dense and α-granules secretion. FcγRIIa triggers an intracellular pathway for platelet activation by phosphorylating the tyrosine kinases Src, Syk, and phospholipase *c* gamma 2 (PLCγ2) (188). Glycoproteins IIb/IIIa (GP IIb/IIIa) and Iba (GPIba), expressed on the surface of activated platelets (202), constitute another receptor category that promotes platelet degranulation and aggregation after binding pathogen components (180, 188, 189, 202). Bacteria have also been found to activate platelets through TLR-2 and TLR-4 (192, 194, 203). Specifically, intracellular signaling of TLR-2 is mediated by PI3 kinase, which activates the Rap1 guanine nucleotide (192). TLR-4 activation in platelets is followed by a complicated intracellular signaling pathway that implicates NFκB. The majority of NFκB signaling proteins are present in platelets, presenting nongenomic effects and resulting in degranulation (203, 204). Moreover, LPS-induced platelet aggregation *in vitro* is dependent on both TLR-4 and TLR-2 platelet receptors as well as on myeloid differentiation factor 88 (MyD88) and cGMP-dependent protein kinase intracellular pathways. Accordingly, LPS accelerated *in vivo* thrombus formation and occlusion in FeCl_3_-injured carotid artery of mice, and this effect was dependent on the MyD88 signaling pathway (194).

**Table 5:** Role of pathogen-activated platelets in immunothrombosis.

Population/cell type	Pathogen/PAMPs	Platelet effect	Method of thrombosis assessment	Effect on thrombosis	Ref
In vitro					
Healthy human blood and PRP	*Mucor circinelloides* NRRL3631	↑ large platelet-spore aggregate formation dependent on FcγRIIA, GPIIb/IIIa, and subsequent Src and Syk tyrosine kinase activation ↑ GPIIb/IIIa, CD62P (P-selectin) expression on platelet surface	Platelet aggregation assessed by LTA	↑ platelet aggregation dependent on TxA2, ADP	(186)
Healthy human PRP	*E. coli* O111	↑ P-selectin in PRP	TF-dependent thrombin generation measured by calibrated automated thrombogram	↑ TF-dependent thrombin generation	(187)
Healthy human platelets	Pam3CSK4, mimetic lipopeptide of Gram+ bacteria, LPS (*E. coli* O111:B4)	↑ NETs in platelet-human neutrophil supernatant dependent on TLR-2 and TLR-4	N/A	N/A	(111)
Healthy human PRP	*S. aureus* Newman, *Streptococcus sanguinis* 133-79, *Streptococcus gordonii* DL1, *Streptococcus oralis* CR834, and *Streptococcus pneumoniae* IO1196	↑ dense and α-granules from platelets dependent on FcγRIIA, GPIIb/IIIa, ADP, TxA2 ↑ tyrosine phosphorylation of FcγRIIA and its downstream signaling partners, Syk and PLCγ2 dependent on GPIIb/IIIa	Platelet aggregation assessed by LTA	↑ platelet aggregation	(188)
Healthy human whole blood	*Streptococcus oralis* CR834	↑ platelet adhesion to immobilized *S. oralis* dependent on GPIbα, under shear rates, mimic arterial and venous conditions	Platelet aggregation assessed by light transmission in a PAP-4 aggregometer	↑ platelet microaggregates	(189)
Healthy human gel-filtered platelet	*Streptococcus oralis* CR834	↑ FcγRIIa-mediated dense granule secretion	Platelet aggregation assessed by LTA	↑ platelet aggregation dependent on ADP	(189)
Healthy human platelets	*S. aureus* Newman	↑ platelet-*S. aureus* adhesion under high shear stress dependent on fibrin	Platelet aggregation assessed by LTA	↑ platelet aggregation dependent on staphylocoagulase, vWbp (*S. aureus* molecules that activate prothrombin)	(190)
Healthy human platelets	LPS (*E. coli* 0111:B4), *S. aureus* α-toxin	↑ TF mRNA through ↑ splicing of TF pre-mRNA dependent on platelet receptor TLR4	TF activity measured by a specific assay (only for LPS)	↑ TF activity	(191)
			Clotting time after suspension in human plasma	↓ clotting time	
Healthy human PRP	*Streptococcus pneumoniae* (12 strains)	↑ dense granule secretion dependent on TLR2 and PI3 kinase-Rap1 guanine nucleotide pathway	Platelet aggregation assessed by LTA	↑ platelet aggregation dependent on TLR2 by five *S. pneumoniae* strains	(192)
Healthy human platelets under iron-limiting conditions	*S. aureus* Newman	↑ platelet-*S. aureus* adhesion-dependent on IsdB (*S. aureus* protein), GPIIb/IIIa, specific interaction of IsdB with GPIIb/IIIa	Platelet aggregation assessed by LTA	↑ platelet aggregation dependent on IsdB	(193)
Healthy human platelets	LPS (*E. coli* 0111:B4)	↑ ATP and ↑ P-selectin in platelet supernatant	Platelet aggregation measured by turbidometric platelet aggregometer under low concentrations of thrombin or collagen	↑ platelet aggregation	(194)
Healthy human PRP	*Streptococcus gordonii* DL1	Firm adhesion of platelets *via* GPIα on Hsa (surface protein of *S. gordonii*) under low shear conditions	Platelet aggregation assessed by LTA	↑ platelet aggregation dependent on GPIIb/GPIIIa	(195)
Healthy human platelets	*Lactococcus lactis* MG1363 expressing SspA and SspB (*S. gordonii* cell wall proteins)	N/A	Platelet aggregation assessed by LTA	↑ platelet aggregation dependent on SspA, SspB, and GPIIb/GPIIIa	(195)
Healthy human PRP	*S. aureus* Newman wild-type and clfA clfB mutants, *Lactococcus lactis* MG1363 expressing *S. aureus* surface proteins ClfA, ClfB, or SdrE	N/A	Platelet aggregation assessed by LTA	↑ platelet aggregation dependent on GPIIb/IIa, ClfA, ClfB, SdrE	(196)
Healthy human PRP	*Streptococcus sanguis* 133-79	N/A	Platelet aggregation assessed by LTA	↑ platelet aggregation-dependent GPIbα and FcγRIIA	(197)
Healthy human platelets	*Porphyromonas gingivalis*	N/A	Platelet aggregation assessed by LTA	↑ platelet aggregation dependent on HRgpA, RgpB proteinases of *P. gingivalis*	(198)
In vivo					
BALB/c mice	Shiga toxin type 2 and *E. coli* O111:B4	↑ plasma platelet-neutrophil aggregates ↑ plasma NETs identified by DNA, histones ↑ plasma vWF (endothelial dysfunction) partially dependent on NETs	N/A	N/A	(199)
C57Bl/6 mice	*S. aureus* α-toxin (strain USA300 LAC, MW2, NRS382, and NRS261)	↑ P-selectin expression by platelets	Platelet aggregation quantified by intravital microscopy	↑ platelet aggregation in the bloodstream	(200)
Ex vivo					
C57BL/6 J mice	CLP sepsis model (polymicrobial)	↑ P-selectin (CD62P), soluble TxB2, sCD40L, and GPIIb/IIIa activation ↑ blood monocyte-platelet and neutrophil-platelet aggregates	Measurement of platelet thrombus volume and formation time in whole blood by a flow assay mimicking microcirculation conditions	Faster adhesion and ↑ formation of platelet thrombus	(173)
Clinical studies					
COVID-19 patients (*n* = 41) and healthy donors (*n* = 17)	SARS-CoV-2	↑ serum P-selectin ↑ neutrophil-platelet aggregates ↑ monocyte-platelet aggregates	Aggregation of isolated platelets from ICU COVID-19 patients and donors after treatment with thrombin, collagen, or 2MeSADP assessed by LTA	↑ platelet aggregation in the COVID-19 group	(201)
Septic patients (*n* = 46) and healthy controls (*n* = 65)	Sepsis (any pathogen)	Spliced TF mRNA in isolated platelets is present only in septic patients	TF activity measured by a specific assay	↑ TF activity in septic patients	(191)

ADP, adenosine diphosphate; ClfA, clumping factor A; ClfB, clumping factor B; CLP, cecal ligation and puncture; E. coli, Escherichia coli; FcγRIIa, low-affinity receptor for the constant fragment (Fc) of immunoglobulin (Ig) G; HRgpA, cysteine proteinases arginine-specific gingipains-R; ICU, intensive care unit; IsdB, iron-regulated surface-determinant B; LPS, lipopolysaccharide; LTA, light transmission aggregometry; 2MeSADP, 2-methylthio-adenosine-5′-diphosphate; PadA, platelet adherence protein A; PAMPs, pathogen-associated molecular patterns; Pam3CSK4, Pam3-cysteine-serine-lysine 4; PRP, platelet-rich plasma; RgpB, cysteine proteinase arginine-specific gingipains-R; S. aureus, Staphylococcus aureus; SARS-CoV-2, severe acute respiratory syndrome coronavirus 2; SdrE, serine-aspartate repeat protein; TF, tissue factor; TxA2, thromboxane A2; TxB2, thromboxane B2; vWbp, von Willebrand factor-binding protein. N/A, non applicable. ↑ (arrow pointing up) means increased. ↓ (arrow pointing down) means decreased.

### Platelet aggregation and thrombus formation in sepsis

All the aforementioned platelet receptors induce immunothrombosis through different pathways after being triggered by PAMPs. The surface of activated platelets promotes thrombin generation and, subsequently, fibrin and clot formation (205). Both dense and α-granules are secreted by pathogen-activated platelets and enhance their aggregation. Specific proteins secreted by platelets are involved in the process of immunothrombosis, such as P-selectin, PF4, vWF from α-granules, and ADP/ATP from dense granules (188). Activated platelets have also been found to release polyphosphate (PolyP), an inorganic polymer that exerts procoagulant activity. *In vitro*, PolyP initiates the contact pathway by FXII activation, resulting in enhanced thrombin generation and clot formation (206). The procoagulant activity of PolyP has also been confirmed in a sepsis mouse model where LPS-induced Polyp is released by platelets, resulting in increased intravascular thrombin activity (108). Protein disulfide isomerase (PDI) is found in the platelet endoplasmic reticulum and is released into circulation by endothelial cells or platelets (157, 207). The pathophysiological mechanism explaining PDI thrombotic effect *in vivo* is not fully elucidated. Activated platelets increase the secretion of PDI, which binds to the GPIIb/IIIa integrin and results in enhanced GPIIb/IIIa-binding affinity. An *in vivo* study suggests that TNF-α activated endothelium induces PDI secretion leading to platelet-neutrophil aggregation through GPIIb/IIIa and αΜβ2 receptors (207). Another possible mechanism has been proposed by an *in vitro* study finding that PDI enhances TF-mediated thrombin generation in peripheral blood mononuclear cells (PBMCs) triggered by LPS (157). Platelet-derived TF levels and activity have also been found to increase *in vitro* after activation by LPS, *E. coli*, or *S. aureus* α-toxin. TLR-4 is one of the platelet receptors mediating TF production by splicing of TF pre-mRNA (187, 191). Indeed, a clinical study confirms that platelets from septic patients present increased TF activity in contrast to healthy ones (191). Furthermore, increased expression of P-selectin and GPIIb/IIIa has been found in a sepsis mouse model and is associated with neutrophil-platelet aggregates and thrombus formation (173). Similarly, a prospective clinical trial found that COVID-19 infection amplifies platelet activation, as indicated by P-selectin, and promotes platelet aggregation (201).

## Targeting immunothrombosis in sepsis

### Clinical evidence on drugs targeting immunothrombosis in sepsis

**Figure 3:**
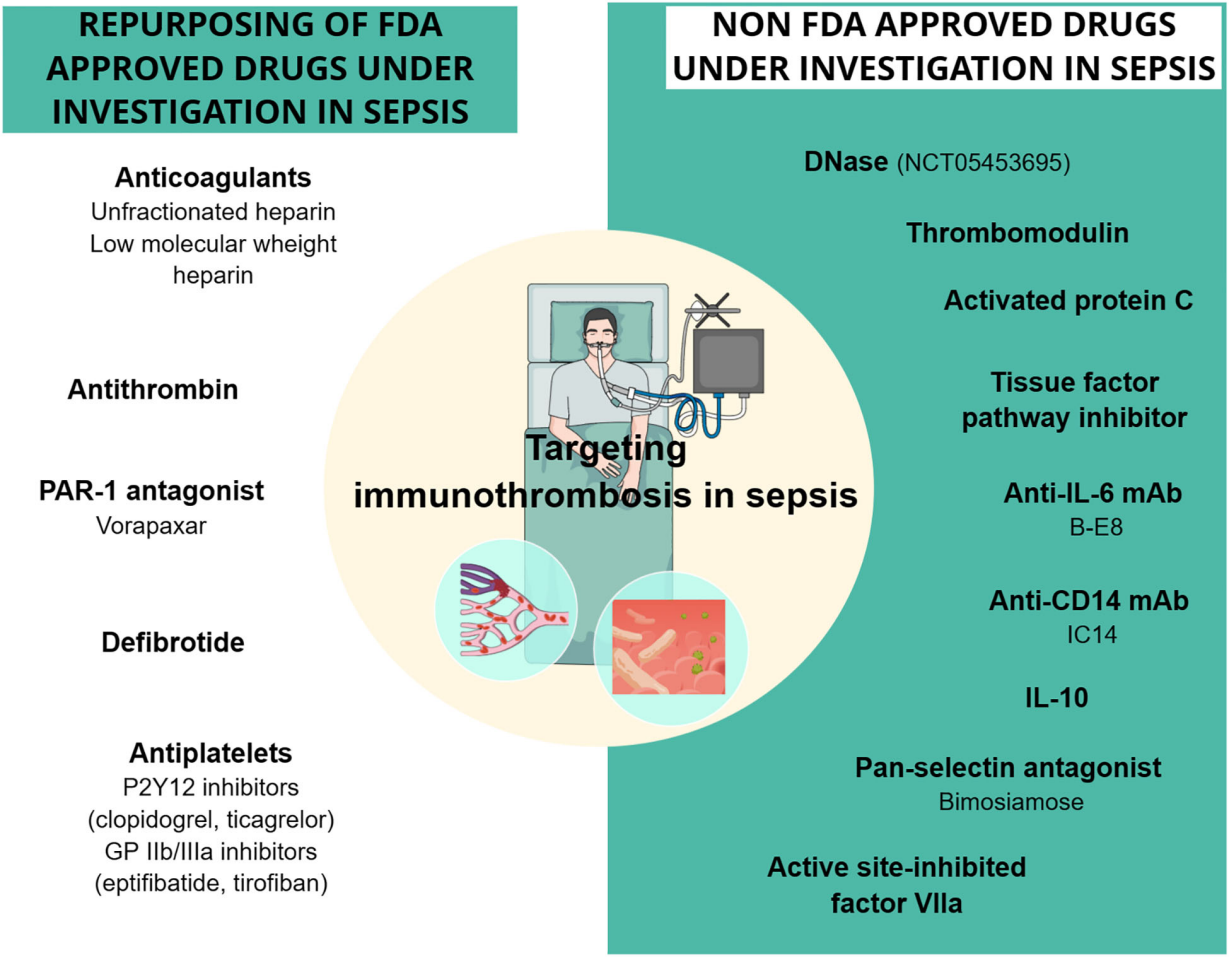
Drugs targeting sepsis-related immunothrombosis in clinical trials. Both FDA-approved and unapproved drugs have been studied in clinical trials for their effect on sepsis-related immunothrombosis. The drugs depicted in the figure showed conflicting results regarding their effect on mortality rates and coagulation parameters in sepsis patients. DNase is the only one with no clinical results yet, as the phase 1 study (NCT05453695) using DNase intervention has recently started. CD14, cluster of differentiation 14; DNase, deoxyribonuclease; GPIIb/IIIa, glycoprotein IIb/IIIa; IL, interleukin; mAb, monoclonal antibody; PAR-1, protease-activated receptor-1.

The efficacy of FDA-approved drugs commonly used in clinical practice, such as heparin, P2Y12, and GPIIb/IIIa inhibitors, has been tested in patients with sepsis. AT, another FDA-approved drug indicated for the prevention of peri-operative and peripartum thromboembolic events in patients with hereditary AT deficiency, has been also assessed (**Table 6**; **Figure 3**) (239). In addition to FDA-approved drugs, a variety of currently non-FDA-approved agents, including recombinant TM, APC, and TFPI, have been evaluated in sepsis (**Table 7**; **Figure 3**). According to the international guidelines for sepsis and septic shock, neither anticoagulant nor antiplatelet medications are recommended for sepsis or DIC treatment, except for low molecular weight heparin (LMWH) or unfractionated heparin (UFH) for VTE prevention (13). On the contrary, AT replacement therapy and recombinant TM are recommended for sepsis-associated DIC in Japan (274). The efficacy of AT in sepsis is controversial, as derived from studies depicted in **Table 6** and meta-analyses (275). Decreased AT plasma levels and DIC presence have been correlated with higher efficacy of AT treatment (221, 234, 275). Furthermore, bleeding risk in patients receiving AT is increased, especially in co-administration with heparin (275). Studies assessing the efficacy of TM have shown more promising results, as TM administration resulted in decreased mortality and was not associated with increased bleeding events in sepsis-induced coagulopathy (276, 277). APC (or drotrecogin alfa activated), despite its antithrombotic effects, is not used in clinical practice as it has not improved survival and has been associated with increased bleeding risk in sepsis (271, 278). Similarly, heparin is not recommended in sepsis management, although some studies have found a beneficial effect on 28-day mortality (279–281). Bleeding events following heparin treatment in sepsis are an important disadvantage of its use (279). Antiplatelet agents, beyond aspirin, have been tested to a lesser extent than anticoagulants, resulting in scarce data regarding their effects on the survival of sepsis patients (215–217). As for aspirin, a recent study showed no effect on mortality in septic patients (282). However, there are multiple underlying mechanisms through which aspirin could affect sepsis, specifically through its known anti-inflammatory properties. As a result, aspirin’s effects on sepsis should not be attributed exclusively to its antithrombotic properties (282). Therefore, drugs targeting immunothrombosis are not approved for clinical use in sepsis due to both inconclusive results for their efficacy and an increased risk for side effects.

**Table 6:** Clinical studies assessing current anticoagulant and antithrombotic drugs in sepsis outcomes.

Study design	Drug	Population	Drug effect	Ref
Randomized controlled trials (RCTs)				
Heparin				
Double-blind RCT	Heparin 70 U/kg/24 h for 5–7 days (*n* = 22) Placebo (n=15)	Patients with sepsis and early DIC (pre-DIC)	No effect on 28-day mortality ↓ MODS, DIC, ventilator days, ICU days, APACHE-II score on day 7	(208)
Double-blind RCT	UFH 500 U/h for 7 days (*n* = 159) Placebo (*n* = 160)	Patients with sepsis	No effect on 28-day and hospital mortality No effect on MOD score and d-dimer levels	(209)
Double-blind RCT	UFH 5,000 U/12 h for 4 days (*n* = 498) Enoxaparin 40 mg/day for 4 days (*n* = 478) Placebo (*n* = 959)	Patients with severe sepsis treated with DrotAA	No effect on any VTE	(210)
RCT	Heparin 2 mg/kg/day of continuous iv infusion (*n* = 55) Placebo (*n* = 64)	Patients with sepsis	↑ platelets ↓ d-dimers, lactic acid plasma levels on days 3, 5, and 10	(35)
Double-blind, phase 4, equivalence design RCT	UFH 5,000 IU/12 h sc for 96 h (*n* = 511) LMWH 40 mg/day sc for 96 h (*n* = 493) Placebo (*n* = 990)	Patients with sepsis treated with DrotAA	↓ 28-day mortality by 3.6% in the heparin group ↑ any bleeding effect in heparin groups (10.8% vs. 8.1%)	(211)
Double-blind RCT	UFH 80 U bolus and 18 IU/kg/h infusion for 6 h (*n* = 10) LMWH 40 U bolus and 15 IU/kg/h infusion for 6 h (*n* = 10) Placebo (*n* = 10)	Healthy humans challenged with LPS (*E. coli*)	↓ TF-positive monocytes, d-dimers, and plasma fibrin polymers in UFH and LMWH group than the placebo ↑ threefold TFPI and FVIIa in UFH and LMWH group than placebo plasma F1 + 2: 10-fold ↑ in the placebo group, twofold ↑ in the LMWH group, no change in the UFH group	(212)
RCT	LMWH 8–15 IU/kg/h (*n* = 15) LMWH 1.5–2 IU/kg/h (*n* = 15)	Patients with sepsis and DIC	↓ d-dimer, TAT complex, F1 + 2 plasma levels on day 7 in the high-dose group	(213)
Open-label RCT	UFH 100 U/kg every 4 h for 12–14 h (*n* = 12) Control group (*n* = 13)	Children with fulminant meningococcemia	No effect on mortality	(214)
P2Y12 inhibitors				
Open-label RCT	Ticagrelor 180 mg/day for 1 week (*n* =10) Control group (*n* = 10)	Healthy humans challenged with LPS (*E. coli*)	↓ platelet-monocyte aggregations (15%) ↓ d-dimer peak plasma levels (48%) ↓ clot density, formation, and lysis assessed by turbidimetric assays ↓ TNF-α (66%), IL-6 (47%), CCL-2 (38%), G-CSF (51%), IL-8 (29%) ↑ IL-10 (54%) No change in hsCRP	(215)
Open-label RCT	Clopidogrel 75 mg/day for 1 week (*n* = 10) Control group (*n* = 10)	Healthy humans challenged with LPS (*E. coli*)	↓ d-dimer peak levels (19%) ↓ TNF-α (60%), IL-6 (28%), CCL-2 (19%) No change in hsCRP	(215)
GP IIb/IIIa inhibitors				
Double-blind, phase 2a RCT	Prostacyclin (iloprost) 1.0 ng/kg/min and eptifibatide 0.5 μg/kg/min infusion for48 h (*n* = 12) Placebo (*n* = 6)	Patients with sepsis	↑ plasma TM, nucleosomes at 6 h only in the placebo group ↓ sE-selectin, sVEGFR1 at 48 h only in the treatment group ↓ platelets at 48 h in the placebo group ↑ platelets at day 7 in the treatment group ↓ fibrin monomers at 48 h, 72 h, and day 5 in the treatment group	(216)
Double-blind RCT	Eptifibatide (180 µg/kg bolus followed by 2 µg/kg/min for 5 h) (*n* = 10) Tirofiban (0.4 µg/kg/min for 30 min followed by 0.1 µg/kg/min for 4.5 h) (*n* = 10) Placebo (*n* = 10)	Healthy male volunteers challenged with LPS (*E. coli*)	No effect on d-dimer, TAT complex, PAP, F1 + 2, TF mRNA, IL-6, CRP, and TNF-α levels	(217)
Antithrombin				
RCT	Ofantithrombin 111 (AT) (*n* = 15) Control group (*n* = 14)	Patients with septic shock and agranulocytosis	↑ 28-day survival in AT group (60% vs. 45%)	(218)
Open-label RCT	AT 30 IU/kg/day for 3 days (*n* = 30) Control group (*n* = 30)	Patients with sepsis, DIC, and AT levels of 50% to 80%	↑ DIC recovery rate in AT group (53.3% vs. 20%) No major bleeding observed	(219)
Double-blind, phase 3 RCT	AT III, 6000 IU/day for 4-day iv infusion (*n* = 805) Placebo (*n* = 811)	Patients with severe sepsis on heparin treatment	No effect on mortality or thromboembolic events	(220)
Double-blind, phase 3 RCT	AT III iv 30,000 IU in total over 4 days (*n* = 305) Placebo (*n* = 294)	Patients with sepsis	↓ 28-day (RR 0.64) and 90-day (RR 0.68) mortality only in the DIC subgroup ↑ major bleeding rates in AT group (7.0% vs. 5.2% in DIC patients) and (9.8% vs. 3.1% for patients without DIC)	(221)
Double-blind blocked RCT	rhAT 130 U/kg loading dose and 130 U/kg/h infusion for 4 h (*n* = 10) rhAT 44 U/kg loading dose and 10 U/kg/h infusion for 4 h (*n* = 10) Placebo (*n* = 10)	Healthy male challenged with LPS (*E. coli*)	↑ aPTT by 10% in the high-dose group vs. placebo ↑ blood clotting time (60%) and clot formation time (40%) in the high-dose group vs. the placebo ↓ F1 +2, TAT complex, and d-dimer in rhAT groups vs placebo ↓ 40% peak IL-6 levels in rhAT groups vs. placebo	(222)
Double-blind phase 3 RCT	AT 6,000 IU bolus followed by 250 IU/h infusion for 4 days (*n* = 17) Placebo (*n* = 16)	Patients with severe sepsis	No effect on aPTT, PT, or whole blood coagulability as assessed by TEG	(223)
RCT	AT infusion for 14 days (*n* = 20) Control group (*n* = 20)	Patients with severe sepsis in surgical ICU	↑ prothrombin, fibrinogen, and prekallikrein plasma concentration in AT group ↑ protein C activity in AT group	(224)
Double-blind, phase 3 RCT	AT III 6,000 IU loading dose and 6,000 IU/day for 4-day iv infusion (*n* = 1,157) Placebo (*n* = 1,157)	Patients with severe sepsis	No effect on 26-, 56-, or 90-day mortality ↑ bleeding incidence in AT group (23.8% vs. 13.5%)	(225)
Double-blind, phase 2 RCT	AT III 1,500 IU/12 h for 5 days (*n* = 20) Placebo (*n* = 22)	Patients with severe sepsis	↓ 30-day mortality by 39%	(226)
RCT	AT III continuous iv infusion for 14 days (*n* = 14) Control group (*n* = 15)	Post-traumatic or postoperative patients with severe sepsis	↓ IL-6, sICAM-1, sE-selectin plasma levels No effect on plasma neutrophil elastase, CRP	(227)
RCT	AT infusion for 14 days (*n* = 20) Control group (*n* = 20)	Patients with severe sepsis in surgical ICU	↓ DIC frequency only in AT group ↑ PaO_2_/FiO_2_ ratio ↓ pulmonary arterial pressure/mean arterial pressure (PAP/MAP) ratio	(228)
Double-blind RCT	ATIII 90 to 120 IU/kg in loading dose, then 90 to 120 IU/kg/day for 4 days (*n* = 17) Placebo (*n* = 18)	Patients with septic shock and DIC	No effect on mortality ↑ DIC resolution on days 2, 4, and 10	(229)
Other				
Double-blind RCT	Vorapaxar (oral PAR-1 antagonist) 10 mg once (*n* = 8) Placebo (*n* = 8)	Healthy humans challenged with LPS	↓ F1 + 2 plasma level maximum increase (27%) ↓ TAT complex levels (22%), PAP complexes (38%), maximum vWF antigen levels (29%), soluble E-selectin maximum increase (30%) ↓peak concentrations TNF-α, IL-6, CRP by 66%, 50% and 23% respectively No effect on soluble P-selectin, TM, PF4	(169)
Double-blind, crossover, blocked-RCT	Defibrotide 6.25 mg/kg infusion for 2 h Placebo	Healthy humans and healthy humans challenged with LPS	↑ PAP complexes, t-PA No effect on TNF-α, IL-6, vWF, E-selectin levels ↓ CRP	(230)
Nonrandomized studies				
Antithrombin				
*Post-hoc* analysis	AT (*n* = 580) Control group (*n* = 618)	Patients with infection-associated DIC received rhsTM	↓ 28-day mortality in the subgroup with low platelets and low AT plasma levels at baseline (HR 0.61) No difference in bleeding adverse drug reactions	(231)
Retrospective cohort study	Recombinant AT gamma 1,800–2,400 IU/day (*n* = 26) Plasma-derived AT 1,500 IU/day (*n* = 23)	Patients with sepsis-induced DIC	No difference in 28-day survival, ventilator-free days	(232)
Prospective, observational study with propensity score matching analysis	AT 30 IU/kg, on the first day and 3,000 IU/day for the next 2 days (*n* = 45) Control group (*n* = 97)	Patients with septic shock, DIC, and AT activity <70%	No effect on 28- and 90-day mortality	(233)
Retrospective cohort study	AT (*n* = 509) Control group (*n* = 524)	Patients with severe sepsis/septic shock and DIC	↓ mortality only in patients with very low AT activity (adjusted HR, 0.603)	(234)
Retrospective propensity score analysis	AT 1,500–3,000 IU/day for 2–4 days (*n* = 518) Control group (*n* = 518)	Patients with mechanically ventilated septic shock and DIC after emergency surgery for perforation of the lower intestinal tract	↓ 28-day mortality by 7.7% ↑ ventilation-free days in AT group (15.2 vs. 13.5)	(235)
Retrospective propensity score analysis	AT 1,563 ± 475 IU/day for 2.7 ± 1.3 days (*n* = 2,194) Matched control group (n=2194)	Patients with severe pneumonia and sepsis-induced DIC	↓ 28-day mortality (OR, 0.85) ↑ ventilation-free days in AT group (9.9 vs. 8.9 days)	(236)
*Post-hoc* analysis	AT III 6000 IU loading dose and 6,000 IU/day for 4-day iv infusion (*n* = 40) Placebo (*n* = 41)	Patients with severe sepsis	No effect on 28- and 90-day mortality No effect on new organ failure incidence ↑ bleeding incidence in AT group (20% vs. 2.4%)	(237)
Prospective interventional trial	AT loading dose and 100–200 IU/kg/day (*n* = 29)	Children with sepsis and coagulopathy	AT, platelet count, fibrinogen, PT, and APTT normalization at 48 h	(238)

APACHE, acute physiology and chronic health evaluation score; aPTT, activated partial thromboplastin time; AT, antithrombin; CCL-2, chemokine (C–C motif) ligand 2; CRP, C-reactive protein; DIC, disseminated intravascular coagulation; DrotAA, drotrecogin alfa activated; E. coli, Escherichia coli; F1 + 2, prothrombin fragment F1 + 2; HR, hazard ratio; ICU, intensive care unit; IL, interleukin; IV, intravenous; kg, kilogram; LPS, lipopolysaccharide; MOD, multiple-organ dysfunction; MODS, multiple-organ dysfunction syndrome; PAI-1, plasminogen activator inhibitor 1; PAP, plasmin-alpha 2–antiplasmin; PF4, platelet factor-4; PT, prothrombin time; RCT, randomized-controlled trial; rhAT, recombinant human antithrombin; rhsTM, recombinant human-soluble thrombomodulin; RR, relative risk; SC, subcutaneous; sICAM-1, soluble intracellular adhesion molecule-1; sVEGFR1, soluble vascular endothelial growth factor receptor 1; TAT, thrombin–antithrombin; TEG, thromboelastography; TF, tissue factor; TFPI, tissue factor pathway inhibitor; TM, thrombomodulin; TNF, tumor necrosis factor; tPA, tissue plasminogen activator; U, units; UFH, unfractioned heparin; VTE, venous thromboembolism; vWF, von Willebrand factor. ↑ (arrow pointing up) means increased. ↓ (arrow pointing down) means decreased.

**Table 7:** Clinical evidence on drugs targeting immunothrombosis in sepsis.

Study design	Drug	Population	Drug effect	Ref
Randomized controlled trials (RCTs)				
Thrombomodulin				
Double-blind, phase 3 RCT	rhsTM 0.06 mg/kg/day iv infusion for 6 days (*n* = 395) Placebo (*n* = 405)	Patients with sepsis-associated coagulopathy	No effect on 28-day mortality ↓ F1 + 2, TAT complexes, d-dimer plasma levels ↑ serious major bleeding event in the rhsTM group (5.8% vs. 4%)	(240)
Open-label RCT	rhTM 380/kg for a maximum of 6 days (*n* = 47) Placebo (*n* = 45)	Patients with sepsis-induced DIC	No effect on survival rate ↑ DIC resolution within 72 h (OR = 2.45) ↑ plasma d-dimer levels relatively decrease from baseline (−0.52 difference from placebo) at day 5	(241)
Double-blind, phase 2b RCT	rhsTM 0.06 mg/kg/day for 6 days (*n* = 371) Placebo (*n* = 370)	Patient with sepsis and suspected DIC	↓ F1 + 2, TAT complexes, d-dimer plasma levels No difference in serious major bleeding events	(242)
Activated protein C				
Double-blind RCT	Protein C concentrate 50 IU/kg and then infusion 3 IU/kg/h for 72 h (*n* = 19) Placebo (*n* = 18)	Patients with severe sepsis or septic shock and a high risk of death and bleeding	No effect on prolonged ICU stay and/or mortality (study early terminated)	(243)
Double-blind, phase 3 RCT	DrotAA 24 μg/kg/h iv infusion (*n* = 846) Placebo (*n* = 834)	Patients with sepsis	No effect on 28-day mortality	(244)
Double-blind RCT	Extended DrotAA 24 μg/kg/h for a maximum of 72 h (*n* = 94) Placebo (*n* = 99)	Patients with severe sepsis and vasopressor-dependent hypotension after a 96-h infusion of standard DrotAA	No effect on vasopressor-dependent hypotension resolution ↓ d-dimers No difference in bleeding events	(245)
Double-blind, phase 3 RCT	DrotAA 24 μg/kg/h iv infusion for 96 h (*n* = 240) Placebo (*n* = 237)	Children with sepsis-induced cardiovascular and respiratory failure	No effect on CTCOFR score of cardiovascular, respiratory, and renal system No effect on 14-, 28-, and in-hospital mortality	(246)
Phase 3 RCT	DrotAA 24 μg/kg/h infusion for 96 h (*n* = 1,316) Placebo (*n* = 1,297)	Patients with severe sepsis and a low risk of death	No effect on 28-day mortality ↑ serious bleeding events until day 28 in the DrotAA group (3.9% vs. 2.2%)	(247)
Double-blind RCT	DrotAA 24 μg/kg/h iv infusion (*n* = 8) Placebo (*n* = 7)	Healthy humans administered endotoxin (*E. coli*) in the lung through bronchoscopy	↓ TAT complex, soluble TF, PAI-1 activity in BALF ↑ protein C in BALF	(248)
Double-blind RCT	Drotrecogin alfa activated 24 mg/kg/h iv infusion for 8 h (*n* = 8) Placebo (*n* = 8)	Healthy humans challenged with endotoxin	No effect on plasma PT, APTT, TM, E-selectin, protein C, PAP complexes, PAI-1, TAT complexes, F1 + 2 No effect on plasma levels of TNF-α, IL-1, IL-6, IL-8, IL-10, and CRP	(249)
Double-blind RCT	DrotAA 24 μg/kg/h iv infusion for 96 h (*n* = 205) Placebo (*n* = 181)	Patients with severe sepsis aged ≥ 75 years	↓ 28-day (RR 0.85) and in-hospital (RR 0.91) mortality ↑ mean number of vasopressor-free days, ventilator-free days, ICU-free days	(250)
Double-blind, phase 3 RCT	DrotAA 24 μg/kg/h iv infusion for 96 h (*n* = 850) Placebo (*n* = 840)	Patients with systemic inflammation and organ failure due to acute infection	↓ death risk (absolute decrease of 6.1%) ↓ plasma d-dimer levels ↑ incidence of serious bleeding in the DrotAA group (3.5% vs. 2%)	(251)
Tissue factor pathway inhibitors				
Double-blind, phase 3 RCT	τifacogin (recombinant TFPI) 0.025 mg/kg/h iv infusion for 96 h (*n* = 880) Placebo (*n* = 874)	Patients with severe sepsis and INR >1.2	No effect on 28-day mortality ↓ TAT complexes and F1 + 2 plasma levels ↑ adverse event with bleeding in the Tifacogin group (24% vs. 19%)	(252)
Single-blind, dose-escalation, phase II RCT	Recombinant TFPI 0.025 (*n* = 80) or 0.05 (*n* = 61) mg/kg/h iv infusion for 4 days Placebo (n=69)	Patients with severe sepsis	↓ 28-day mortality in the TFPI group (30% vs. 38%) No difference in organ dysfunction Scores ↓ TAT complex plasma levels ↓ IL-6 plasma levels	(253)
Double-blind, crossover RCT	Recombinant TFPI 0.0125 mg/kg and then 0.05 mg/kg/h for 6 h Recombinant TFPI 0.05 mg/kg and then 0.2 mg/kg/h for 6 h (*n* = 8 high and low doses) Placebo (n=8)	Healthy humans challenged with LPS (*E. coli*)	↓ TAT complex plasma levels and aPTT only in the high-dose group ↓ F1 + 2 plasma levels and PT both in high- and low-dose group No change in plasma levels of TNF, IL-6	(254)
Other				
Double-blind two-way crossover RCT	Bimosiamose (pan-selectin antagonist) 30 mg/kg infusion Placebo (*n* = 16) washout period 6 weeks	Healthy male volunteers challenged with LPS (*E. coli*)	No effect on F1 + 2, TAT complex, TF mRNA, IL-6, TNF-a plasma levels	(255)
Double-blind RCT	Monoclonal anti-IL-6 Ab (B-E8) (*n* = 12) Placebo (*n* = 12)	Healthy male volunteers challenged with LPS (*E. coli*)	↓ CRP No effect on F1 + 2, TAT complex, TF mRNA, IL-10, TNF-a, PAP complex, sE-selectin plasma levels	(256)
Double-blind RCT	IC14 (recombinant chimeric anti-CD14 mAb) 1 mg/kg iv once (*n* = 8) Placebo (*n* = 8)	Healthy male volunteers challenged with LPS (*E. coli*)	↓ TAT complex, soluble fibrin plasma levels No effect on F1 + 2 plasma levels, TF expression by whole blood cells ↓ tPA, PAI-1, PAP complexes plasma levels	(257)
Double-blind RCT	Active site-inhibited factor VIIa (FFR-recombinant factor VIIa) 400 μg/kg bolus infusion (*n* = 6) Placebo (*n* = 6)	Healthy male volunteers challenged with LPS (*E. coli*)	↓ F1 + 2, TAT complex plasma, soluble fibrin, d-dimer levels	(258)
Double-blind, crossover RCT	Recombinant human IL-10 (*n* = 8) 25 μg/kg Placebo (*n* = 8)	Healthy male volunteers challenged with LPS (*E. coli*)	↓ d-dimer, t-PA, PAP complexes, PAI-1, TAT complexes, F1 + 2 (plasma levels)	(259)
Nonrandomized studies				
Thrombomodulin				
Retrospective study	rhsTM 80 U/kg/day for 6 days (*n* = 123)	Patients with sepsis-induced DIC	↓ DIC score, PT, FDP ↑ platelet count	(260)
*Post-hoc*, subgroup analysis	rhsTM 0.06 mg/kg/day for 6 days (*n* = 395) Placebo (*n* = 405)	Patients with sepsis-associated coagulopathy	No effect on 28-day all-cause mortality	(261)
Retrospective cohort study	rhsTM 150 or 380 U/kg/day until DIC resolution (*n* = 17) Placebo (*n* = 25)	Patients with sepsis-induced DIC caused by acute cholecystitis	No effect on PT, fibrin degradation products (FDP) ↑ platelets on days 3 and 7 ↑ 26.1% DIC resolution on day 7	(262)
Retrospective propensity score analysis	rhTM (*n* = 356) Control group (*n* = 824)	Sepsis patients with severe respiratory failure	↓ hospital mortality in the rhTM group (35.6% vs. 49.7%) ↓ ICU mortality in the rhTM group (22.1% vs 36.2%) No difference in bleeding adverse events	(263)
Retrospective, propensity score analysis	rhsTM 130 IU/kg/day (*n* = 452) Placebo (*n* = 452)	Patients with DIC and severe sepsis or septic shock	↓ in-hospital all-cause mortality (OR, 0.75) No difference in bleeding complications	(264)
Prospective observational study	AT 1,500 IU/day and rhTM 0.02 mg/kg/day (median doses) (*n* = 78) AT 1,500 IU/day (median dose) (*n* = 51)	Patients with severe sepsis and DIC	↓ mortality in AT + rhTM group (15.4% vs. 29.4%) ↑ platelets in AT + rhTM group on day 7 ↓ d-dimers in AT + rhTM group on day 7 No difference in the incidence of bleeding complications	(265)
Retrospective propensity score analysis	rhsTM 0.06 mg/kg/day iv infusion for 6 days (*n* = 68) Control group (*n* = 94)	Patients with sepsis-induced DIC	↓ in-hospital (HR, 0.45), ICU (HR, 0.49) mortality ↓ 28-day (HR, 0.48), 60-day (HR, 0.47), and 90-day (HR, 0.45) mortality No difference in the incidence of bleeding-related adverse events	(266)
Prospective interventional trial	rhsTM 0.06 mg/kg/day iv infusion for 6 days (*n* = 20) Control group (*n* = 45)	Patients with sepsis-induced DIC who required ventilatory management	↓ 28-day mortality (HR, 0.303) ↓ SOFA score ↑ platelet count recovery ↑ FDP reduction from baseline	(267)
Activated protein C				
Retrospective, propensity-matched, cohort study	rhAPC (*n* = 311) Control (*n* = 622)	Patients with septic shock	↓ 30-day mortality (HR, 0.72) ↑ median ICU length of stay in the rhAPC group (8 vs. 7 days) No difference in ventilator-free days	(268)
*Post-hoc* analysis of a double-blind, phase 3 RCT	DrotAA 24 μg/kg/h iv infusion for 96 h (*n* = 240) Placebo (*n* = 237)	Children with sepsis-induced cardiovascular and respiratory failure	↓ d-dimer on day 1 and TAT complex on days 1 to 4 No effect on PCT, TNF-α, IL-1β, IL-6, IL-8, IL-10, IL-12	(269)
Prospective interventional trial	Protein C concentrate 100 IU/kg iv bolus followed by doses of 50 IU/kg/6 h for 72 h	Neonates with sepsis-induced coagulopathy	↓ PT, Aptt at 48 h ↑ AT activity at 48 h ↑ PaO_2_/FiO_2_, MAP ↓ CRP at 72 h	(270)
Prospective interventional trial	Protein C concentrate 50 IU/kg bolus followed by continuous infusion of 3 IU/kg/h for 72 h	Patients with severe sepsis or septic shock and two or more organ failure after cardiac surgery	↓ ISTH DIC score at 30 h ↓ PT, aPTT, TAT complexes ↑ AT activity ↓ plasma levels of IL-6, IL-8, and IL-10 at 6, 42, and 18 h, respectively	(271)
*Post-hoc* analysis	DrotAA 24 μg/kg/h iv infusion for 96 h (*n* = 850) Placebo (*n* = 840)	Patients with systemic inflammation and organ failure due to acute infection	↑ resolution of cardiovascular (HR, 1.19) and respiratory failure (HR, 1.41)	(272)
Other				
*Post-hoc* subgroup analysis	Any anticoagulant (*n* = 1,247) Control group (*n* = 1,416)	Patients with sepsis	↓ in-hospital mortality only in the DIC subgroup (HR, 0.609)	(273)

aPTT, activated partial thromboplastin time; AT, antithrombin; BALF, bronchoalveolar lavage fluid; CRP, C-reactive protein; CTCOFR, Composite Time to Complete Organ Failure Resolution; DIC, disseminated intravascular coagulation; DrotAA, drotrecogin alfa activated; E. coli, Eshcerichia coli; F1 + 2, prothrombin fragment F1 + 2; FDP, fibrin/fibrinogen degradation products; HR, hazard ratio; ICU, intensive care unit; interleukin; ISTH, International Society on Thrombosis and Haemostasis; iv, intravenous; LPS, lipopolysaccharide; mAb, monoclonal antibody; MAP, mean arterial pressure; OR, odds ratio; PAI-1, plasminogen activator inhibitor 1; PAP, plasmin-alpha 2–antiplasmin; PCT, procalcitonin; PT, prothrombin time; RCT, randomized-controlled trial; rhAPC, recombinant human-activated protein C; rhTM, recombinant human thrombomodulin; rhsTM, recombinant human-soluble thrombomodulin; RR, relative risk; sE-selectin, soluble E-selectin; SOFA, sequential organ failure assessment; TAT, thrombin–antithrombin; TF, tissue factor; TFPI, tissue factor pathway inhibitor; TNF-α, tumor necrosis factor-alpha; tPA, tissue plasminogen activator; U, units. ↑ (arrow pointing up) means increased. ↓ (arrow pointing down) means decreased.

### Preclinical evidence on potential molecules targeting immunothrombosis in sepsis

**Figure 4:**
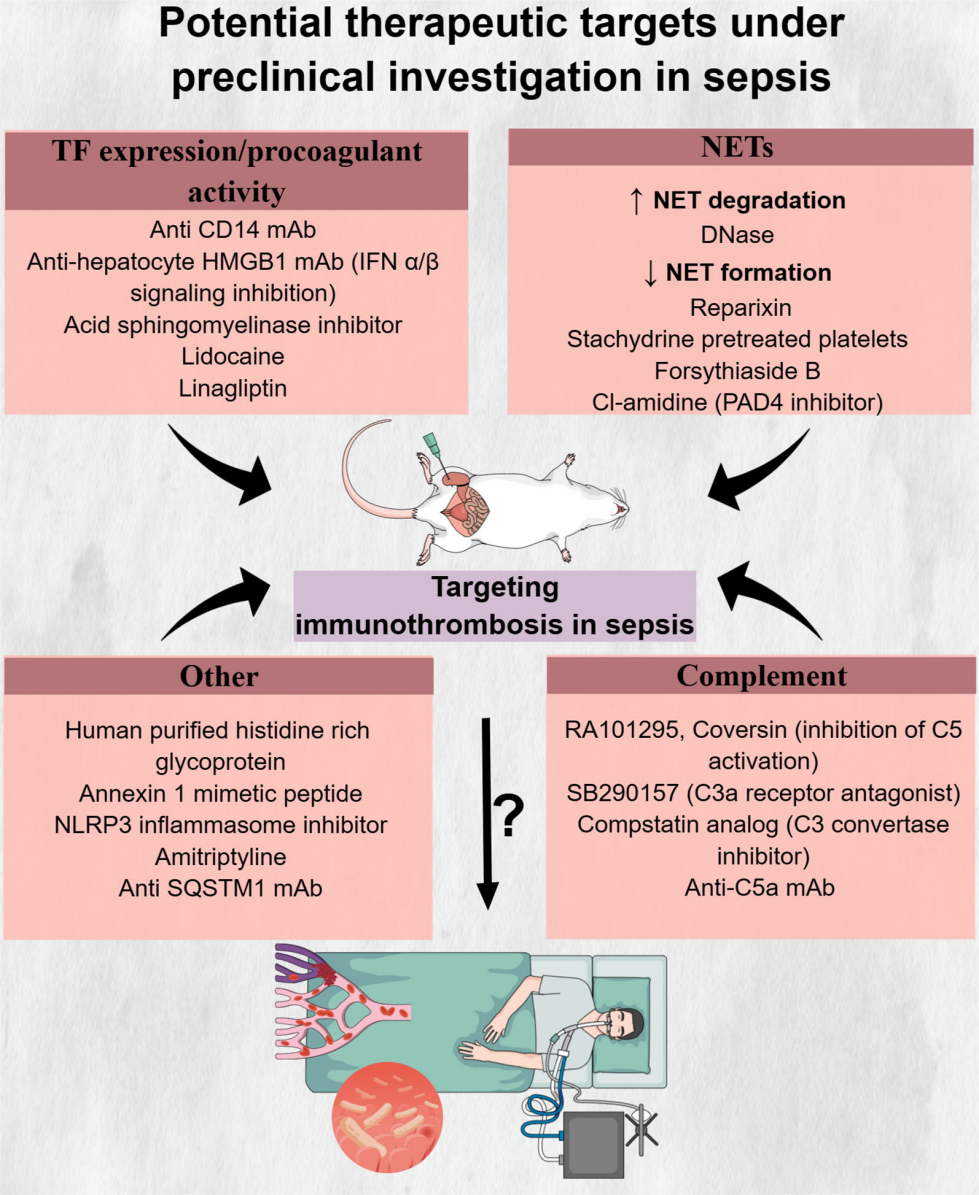
Potential therapeutic targets under preclinical investigation in sepsis. Inhibitors of innate immune components, like neutrophil extracellular traps and complement components C5 and C3, drugs interfering with tissue factor formation and procoagulant activity as well as molecules with multiple actions, have shown promising results in reducing immunothrombosis in preclinical sepsis models. However, the safety, tolerability, and effectiveness of these drugs in abrogating the deleterious effects of immunothrombosis in patients with sepsis are still unknown. C5, complement 5; C3, complement 3; CD14, cluster of differentiation 14; DNase, deoxyribonuclease; HMGB1, high mobility group box 1; IFN, interferon; mAb, monocloncal antibody; NETs, neutrophil extracellular traps; NLRP3, NLR family pyrin domain containing 3; PAD4, protein arginine deiminase; SQSTM1, sequestosome 1; TF, tissue factor.

The general failure of clinical trials to identify pharmaceutical agents with an acceptable risk-to-benefit ratio poses the need for discovering new molecules that could mitigate the deleterious effects of immunothrombosis in sepsis. A variety of such molecules have been examined in sepsis animal models, showing promising results, as depicted in **Table 8** and **Figure 4**. Administration of histidine-rich glycoprotein (HRG), a plasma protein found to be significantly depleted in septic conditions, attenuated immunothrombi formation in the lungs of septic mice (294). Reduced neutrophil adherence to endothelium due to shape changes and decreased NET generation, as well as reduced endothelial cell activation, all mediated by HRG, were suggested to be among the potential mechanisms responsible for this effect (294). Another study investigated the beneficial role of Annexin A1, an anti-inflammatory protein found in low levels in patients with sepsis, on LPS-induced thrombosis (287, 299). Interestingly, Annexin A1 mimetic peptide attenuated platelet aggregation and resulted in delayed and reduced microthrombi formation in the cerebral microcirculation (287).

**Table 8:** Preclinical evidence of novel drugs targeting immunothrombosis in sepsis.

Animal population	Sepsis cause	Intervention	Effect on thrombosis	Ref
C57BL/6 J mice	CLP	Stachydrine pretreated platelets	↓NET-mediated lung thrombosis ↓ microthrombus formation in central veins in the liver	(136)
Sprague–Dawley rats	CLP	Forsythiaside B or DNase 1 or Cl-amidine (PAD4 inhibitor)	↓ NET formation *via* PAD4 inhibition ↑ platelets, ↓ PT, APTT, ↑ fibrinogen ↓ vWF, TM, P-selectin, PAI-1, PAF, and TAT	(283)
C57BL/6J mice	LPS (*Salmonella typhosa*)	Linagliptin (dipeptidyl peptidase-4 inhibitor)	↓ pulmonary microvascular thrombosis	(59)
C57/BL6 mice	CLP or *E. coli*	Reparixin (inhibitor of CXCR1/2 signaling)	↓ NET formation in plasma and lungs, ↓ fibrin deposition within the lungs	(284)
Baboon	*E. coli*	Anti-CD14 mAb	↓ PT, APTT ↓ FVII-AT complexes ↓ APC-α1-antitrypsin complexes ↓ TF mRNA in circulating blood cells and lung ↓ PAI-1, ↑ PAP, ↑ d-dimers	(285)
CD-1 mice	LPS	Lidocaine	↓ TF expression in plasma and lungs ↓ pulmonary thrombosis	(286)
C57BL/6 mice	LPS (*E. coli* 0111:B4)	Annexin A1 (AnxA1) mimetic peptide	Delayed thrombus formation in cerebral venules Mitigation of thrombus formation in cerebral arterioles and venules ↓ bleeding time ↓ velocity of platelet aggregation formation	(287)
C57Bl/6 mice	LPS (*E. coli* 0111:B4)	DNase	↓ active thrombin generation within liver sinusoids	(138)
Sprague–Dawley rats	CLP	MCC950 (specific NLRP3 inhibitor)	↓ activated platelets, ↓ serum PF4 ↓ plasma Endocan levels	(288)
C57BL/6J mice	LPS	Antihepatocyte HMGB1 mAb	↓ intravascular thrombin and ↓platelet aggregation in liver microvasculature, ↓ plasma TAT complexes and PAI1	(289)
C57BL/6J mice	*E. coli* or *S. pneumoniae* or CLP	Anti-SQSTM1 mAb	↓ thrombocytopenia, ↓ PT and APTT ↑ fibrinogen, ↑ d-dimers	(290)
Kunming mice	LPS (*E. coli* O55:B5)	Embelin (PAI-1 inhibitor)	↓ circulating TF levels, ↓ microthrombi formation in lungs, ↓ thrombocytopenia, APTT, PT, and fibrinogen consumption, ↑ bleeding time	(291)
CF-1 outbred mice	CLP	Amitriptyline (antidepressant)	↓ carotid occlusion time ↓ clotting formation time	(292)
C57BL/6J) mice	LPS (*E. coli* O111:B4)	Desipramine or imipramine (acid sphingomyelinase inhibitors)	↓ plasma TAT complexes ↓ TF procoagulant activity in isolated PBMCs and microvesicles	(162)
C57Bl/6J mice	LPS (*E. coli* 0111:B4)	DNase	↓ intravascular thrombin activity ↓ microvascular occlusion in liver sinusoids	(108)
Baboon	*E. coli*	RA101295 (inhibition of C5 activation)	↓ PT, APTT ↓ FXII consumption, ↓ FXIa-AT and FVIIa-AT complexes ↓ TAT complexes, t-PA, PAI-1, PAP complexes, d-dimers, FDP	(91)
C57BL/6J mice	LPS (*Salmonella typhosa*)	Linagliptin (dipeptidyl peptidase-4 inhibitor) or liraglutide (GLP-1 receptor agonist)	↓ thrombocytopenia, ↓ pulmonary microvascular thrombosis	(293)
C57BL/6N mice	CLP	Human-purified histidine-rich glycoprotein (HRG)	↓ immunothrombi (composed of neutrophils, platelets, and fibrin) in the lung vasculature ↓ neutrophils and aggregated platelets attached to ECs in venules ↓ NETs Platelet count restoration ↓ APTT and PT	(294)
Norwegian landrace pigs	*E. coli* (strain LE392)	Coversin (inhibition of C5 activation) and anti-CD14 mAb	↓ TF and PAI-1 mRNA in the spleen	(295)
C57Bl/6 mice	CLP	Delayed (4 or 6 h after CLP) or early (2 h after CLP) DNase infusion	↓ plasma TAT complexes levels only after delayed DNase infusion	(296)
Norwegian landrace pigs	*E. coli* (strain LE392) induced sepsis	Coversin (inhibition of C5 activation) and anti-CD14 mAb	↓ TF expression, TAT complexes (↓ with combined coversin and anti-CD-14> coversin alone) ↓ PAI-1 (only with combined coversin and anti-CD-14)	(297)
C57BL/6 mice	LPS and Shiga toxin (*E. coli* O157:H7)	SB290157 (C3a receptor antagonist)	↓ glomerular fibrin(ogen) deposition, ↓ platelet clumps, ↓ glomerular TM loss	(82)
Pigs	LPS (*E. coli* 0111:B4) or live *E. coli* (strain LE392)	Anti-CD14 mAb	↓ TAT complexes levels, ↓ PAI-1	(298)
Baboon	*E. coli*	Compstatin analog (C3 convertase inhibitor)	↓ thrombocytopenia ↓ fibrinogen consumption and FDPs ↓ accumulation of platelets in the lung and on the surface of large vessels ↓ microvascular thrombosis in the lungs	(93)
Rats	CLP	Anti-C5a	↓ APTT and PT ↑ FVII:C, ↓ fibrinogen, ↑ AT, ↑ plasminogen, ↓ t-PA, ↓ PAI, ↓ TAT complex, ↓ d-dimers ↓ thrombocytopenia	(94)

APC, activated protein C; APTT, activated partial thromboplastin time; AT, antithrombin; CD14, cluster of differentiation 14; CLP, cecal ligation puncture; CS, cecal slurry; DNase, deoxyribonuclease; E. coli, Escherichia coli; ECs, endothelial cells; FDP, fibrin degradation products; GLP, glucagon-like peptide; HMGB1, high-mobility group box-1 protein; ip, intraperitonealy; LPS, lipopolysaccharide; mAb, monoclonal antibody; NETs, neutrophil extracellular traps; NLRP3, NLR family pyrin domain containing 3; PAF, platelet-activating factor; PAD 4, peptidylarginine deiminase 4; PAI-1, plasminogen activator inhibitor; PAP, plasmin-alpha 2-antiplasmin; PF4, platelet factor 4, PT, prothrombin time; rhTM, recombinant human thrombomodulin; SQSTM1, sequestosome 1; TAT, thrombin–antithrombin; TF, tissue factor; TFPI, tissue factor pathway inhibitor; TM, thrombomodulin; vWF, von Willebrand factor. ↑ (arrow pointing up) means increased. ↓ (arrow pointing down) means decreased.

A variety of inhibitory molecules targeting pathways involved in TF expression in immune or endothelial cells or in TF procoagulant activity have also been proposed. Disrupting TLR4-mediated LPS signaling through cluster of differentiation 14 (CD14) inhibition in immune cells was one potential pathway to target. Specifically, CD14 is a protein responsible for LPS transfer to its cell surface receptor complex, TLR4/myeloid differentiation factor 2 (MD-2) (a co-receptor of TLR4) (285). Inhibitory monoclonal antibody (mAb) against CD14 led to decreased activation of the extrinsic coagulation pathway through suppression of TF expression in leukocytes while also enhancing fibrinolysis through decreasing plasma PAI-1 (285). Apart from anti-CD14 mAb, lidocaine could also inhibit LPS-induced TF expression in monocytes through upregulation of suppressor of cytokine signaling 3 (SOCS3), which in turn downregulated the TLR4-TF pathway (286). Another potential pathway that could serve as a therapeutic target for mitigating immunothrombosis was shown to be type 1 interferon (IFN-1)-induced upregulation of TF procoagulant activity. Type 1 IFNs are cytokines that increase in response to bacterial infection and are characterized by antimicrobial properties. Inhibition of the expression of IFN-1 receptors or downstream effectors (e.g., HMGB1) in macrophages reduced gram-negative bacteria-induced DIC (289). TF procoagulant activity was also found to be decreased by acid sphingomyelinase inhibitors. In response to LPS, acid sphingomyelinase translocates to the cell membrane and hydrolyzes sphingomyelin, which, in resting conditions, maintains TF in an encrypted form with minimal procoagulant activity. Therefore, inhibition of acid sphingomyelinase inhibitors could maintain sphingomyelin levels on cell membranes despite LPS stimulation, resulting in decreased TF activity and reduced TAT levels in a sepsis mouse model (167). Linagliptin, a dipeptidyl peptidase-4 inhibitor used for diabetes treatment, was also found to ameliorate LPS-induced pulmonary thrombosis in mice *via* decreased endothelial TF expression. This effect was shown to be mediated through linagliptin-induced activation of the Akt/eNOS pathway and subsequent NO production (59). Noteworthy, linagliptin or the glucagon-like peptide-1 (GLP-1) analog, liraglutide, were shown to have further inhibitory effects on platelet thrombin generation and platelet aggregation *via* the cyclic AMP (cAMP)-protein kinase A (PKA) signaling pathway (293). Among other clinically approved drugs, amitriptyline, a tricyclic antidepressant, was also capable of reducing thrombosis through TNF-α level reduction (292).

The NLRP3 inflammasome, a complex responsible for IL-1β and IL-18 release and a crucial mediator of pyroptosis, became activated in platelets in a sepsis rat model and correlated with lung and renal injury (300, 301). Blockage of NLRP3, using a selective inhibitor, led to decreased platelet activation and offered protection from organ dysfunction in rats with sepsis (288). Another potential therapeutic target was shown to be SQSTM1, a stress response protein that was released from monocytes and macrophages upon LPS stimulation (290). Interestingly, an inhibitory antibody against SQSTM1 attenuated DIC in bacteria-induced sepsis (290).

Promising results have also been demonstrated in preclinical studies examining the effect of NET inhibition on sepsis-induced thrombosis (108, 138, 296). DNase is an enzyme capable of digesting DNA. Two types of DNases have been found: DNase 1, which degrades free DNA, and DNase 1-like 3 (DNase1L3), which degrades DNA bound to proteins, such as histones (140). Patients with sepsis were shown to have decreased levels of DNase 1 compared to healthy controls (302). Moreover, mice with a combined deficiency of DNase1 and DNase1L3 demonstrated increased thrombosis in pulmonary vessels compared to mice capable of producing at least one of these two enzymes when both were infected either with LPS or heat-killed *E. coli* (140). Exogenous DNase administration promoted NET degradation and resulted in reduced intravascular thrombin activity as well as decreased microthrombus formation in liver sinusoids (108). The timing of the DNase infusion seems to affect its beneficial effect, with delayed infusion resulting in a significant decrease in TAT complexes compared to the insignificant effect observed with early infusion (296). Importantly, DNase administration, aiming to decrease LPS-induced NETosis, prior to *S. aureus* infection, did not negatively affect pathogen capture and clearance (138). However, the impact of DNase infusion after bacterial infection was not examined in that study. In contrast, mice treated with DNase at seven consecutive time points, beginning 1 h after cecal ligation and puncture (CLP), showed increased bacterial dissemination and higher serum IL-6 levels 6 h after CLP and higher mortality at 24 h (303). However, it should be noted that pathogens’ spread and levels of systemic inflammation 24 h after CLP, as well as overall survival, did not increase in DNase-treated mice compared to controls (303). Besides the evaluation of DNase treatment in preclinical sepsis models, a clinical trial (NCT05453695) is going to assess intravenous DNase safety and tolerability as well as its effect on organ dysfunction, duration of ICU hospitalization, mortality, and blood coagulation in patients with sepsis (304). Regarding inhibition of NET formation, reparixin, an inhibitor of interleukin-8 CXC chemokine receptor type 1/2 (CXCR1/2), resulted in decreased NET formation and subsequently reduced fibrin deposition in the lungs of septic mice (284). Similarly, infusion of platelets pretreated with stachydrine, an herbal product, attenuated NET formation, platelet–neutrophil interaction, and subsequent thrombosis (136).

Complement activation, despite being an essential component of the innate immune response, is associated with increased mortality in sepsis (305, 306), while it also contributes to immunothrombosis (307, 308). Blockade of C3a or C5a led to decreased DIC in *E. coli* or (CLP)-induced sepsis animal models (91, 93, 94). Noteworthy, combined inhibition of C5 activation and CD14 attenuated more robustly hypercoagulability in sepsis (295, 297).

## Conclusion

Immunothrombosis is a key pathophysiological mechanism of sepsis, arising from the complex interplay between innate immune and endothelial cells on one side and platelets and the coagulation cascade on the other. Although initially beneficial for the host, uncontrolled and systemic activation of this process in sepsis can lead to thrombotic and bleeding complications, ranging from subclinical abnormalities in coagulation tests to severe clinical manifestations, such as DIC. Endothelial dysfunction, characterized by glycocalyx degradation, increased vascular permeability, as well as proinflammatory and procoagulant properties of endothelial cells, further facilitates immunothrombosis progression. Despite the high burden of coagulation disorders that have a negative impact on sepsis patients’ prognosis, there is no effective treatment aiming to target the immunothrombotic process. Although a variety of anticoagulants and antiplatelet drugs have been evaluated in clinical trials, their beneficial effects are inconsistent, and they are also characterized by a high rate of bleeding complications. However, there is still ongoing research assessing the effect of new molecules on thrombosis in sepsis, with agents targeting intracellular inflammatory pathways, NET formation, and complement components demonstrating promising results. Future studies are called to unravel the pathophysiologic mechanisms involved in immunothrombosis in sepsis, to identify potential prognostic biomarkers, to develop risk scores to predict the outcomes of sepsis, and to test novel therapeutics against immunothrombosis in sepsis.

## Author contributions

EM and EA researched data for the article, contributed to the interpretation of the published studies, and drafted all display items. KSte developed the concept, discussed the content, jointly conceived the display items of the article, and edited the article. ST-C, BEV, AG, and KSta provided conceptual advice, participated in the interpretation of the data, and edited the manuscript. All authors contributed to the article and approved the submitted version.
